# Supernatants of Water Extraction–Ethanol Precipitation from Roots of *Rosa davurica* Pall. Ameliorate Ulcerative Colitis in Mice: From Gut Microbes to Metabolites

**DOI:** 10.3390/ph19091372

**Published:** 2026-08-30

**Authors:** Lihao Wang, Wuyou Gao, Yuesong Xiao, Ting Yang, Jingwei Wang, Yanping Sun

**Affiliations:** Key Laboratory of Basic and Application Research of Beiyao, Heilongjiang University of Chinese Medicine, 24 Heping Road, Xiangfang District, Harbin 150040, China

**Keywords:** ulcerative colitis, *Rosa davurica* Pall., NF-κB/NLRP3-related genes, gut microbiota, metabolomics

## Abstract

**Background:** Safe treatment options for ulcerative colitis, a chronic relapsing inflammatory bowel disease, remain limited, and the bioactive fraction of *Rosa davurica* Pall. root associated with its protective activity against Ulcerative colitis (UC) has not been characterized. **Methods:** This study evaluated the protective effects of the total root aqueous extract (WRP) and two process-defined fractions prepared by ethanol precipitation: the supernatant fraction (SRP) and crude polysaccharide precipitate fraction (PRP). These fractions were administered concurrently with Dextran sulfate sodium (DSS) during acute colitis induction in mice. Assessments included disease activity index, colon histopathology, intestinal barrier proteins, RT-qPCR analysis, fecal metabolomics, 16S rRNA sequencing, and UHPLC-MS/MS. **Results:** Compared to PRP, SRP significantly alleviated DSS-induced weight loss, colon shortening, perianal bleeding, and histological injury. SRP also reduced the mRNA levels of TNF-*α*, IL-6, IL-1*β*, MPO, NF-*κ*B, NLRP3, GSDMD, and IL-18. SRP treatment increased Claudin-1 and ZO-1 protein expression, whereas Occludin expression showed no significant change. SRP treatment was associated with shifts in fecal metabolic profiles and gut microbiota composition in DSS-treated mice, including a change in the *Firmicutes/Bacteroidota* ratio toward the CON group pattern. UHPLC–MS/MS profiling resulted in the tentative annotation of 68 nonredundant constituents in SRP. **Conclusions:** Under the present experimental conditions, SRP demonstrated protective activity in DSS-induced acute colitis and represents a potentially important bioactive fraction of the *R. davurica* root aqueous extract. Its protective effects were accompanied by reduced inflammatory gene expression, partial enhancement of barrier-associated protein expression, and alterations in gut microbiota composition and fecal metabolic profiles.

## 1. Introduction

Worldwide, the incidence of ulcerative colitis (UC), a chronic, nonspecific inflammatory bowel disease, continues to rise [1]. Clinically, UC is primarily characterized by abdominal pain, diarrhea, hematochezia, and anemia. With a typically relapsing–remitting course, UC significantly compromises patients’ quality of life and can become life-threatening [2]. Moreover, both the incidence and prevalence of UC have increasingly shifted toward younger populations [3]. Although the precise pathogenesis of UC remains incompletely understood, it is widely accepted to involve dietary factors, genetic susceptibility, immune dysregulation, alterations in gut microbiota, infections, and systemic inflammation [4]. Current clinical strategies for managing UC emphasize controlling acute flares, alleviating symptoms, reducing recurrence, promoting ulcer healing, and preventing complications. However, many patients experience frequent relapses and suboptimal therapeutic responses. Conventional treatments entail considerable adverse effects: aminosalicylates may induce severe gastrointestinal reactions and renal injury [5,6]; glucocorticoids can result in osteoporosis and osteonecrosis [7]; anti-TNF agents may lead to infections, drug-induced lupus, and congestive heart failure [6]; and while promising, JAK inhibitors come with high costs and risks of immunosuppression, thrombosis, hypercholesterolemia, and even cancer [8,9,10]. Thus, an urgent need persists for safer and more effective alternatives.

Traditional Chinese herbal medicines have shown potential in alleviating UC symptoms, improving epithelial barrier function, maintaining Th17/Treg balance, and modulating gut microbiota [11,12]. *Rosa davurica* Pall. (*R. davurica*) is a perennial deciduous shrub of the Rosaceae family, found in northern China, Korea, eastern Siberia, and southern Mongolia [13]. As both a medicinal and edible plant, various parts of *R. davurica*, including its fruits and roots, have been traditionally utilized. Notably, the fruits have a documented history of culinary use in China, including incorporation into beverages and food preparations, with a local food-safety standard for *R. davurica* (DBS23/017-2022) established in Heilongjiang Province [14,15]. The roots contain diverse constituents, such as polyphenols, fatty acids, and tannin-related compounds, exhibiting antioxidant, anti-diabetic, hepatoprotective, and *α*-glucosidase inhibitory activities [14,16]. However, the protective effects of *R. davurica* root against UC have not been reported, and no in vivo study has assessed *R. davurica* extracts in experimental colitis. As an initial investigation, aqueous and 75% ethanol extracts prepared from the flowers, fruits, and roots of *R. davurica* were evaluated in DSS-induced colitis mice. Among the tested extracts, the root aqueous extract demonstrated the most consistent protective profile, significantly improving the disease activity index (DAI) and ameliorating colon shortening, histopathological injury, and elevated colonic pro-inflammatory cytokine levels. Consequently, it was selected for further activity-guided evaluation of its ethanol-precipitation fractions, with detailed screening results provided in the Supplementary Materials. The total root aqueous extract was designated WRP, while a parallel aqueous extract prepared under the same conditions underwent ethanol precipitation to yield a supernatant fraction (SRP) and a crude polysaccharide precipitate fraction (PRP). Thus, WRP denoted the unfractionated aqueous extract, while SRP and PRP represented the process-defined supernatant and precipitate fractions, respectively. This design facilitated the assessment of whether protective effects associated with the root aqueous extract were primarily retained in the supernatant or precipitate fraction following ethanol precipitation. Under the present experimental conditions, SRP exhibited protective effects across several endpoints that mirrored improvements observed with WRP, whereas PRP did not demonstrate evident protection. These findings suggest that SRP is a potentially important bioactive fraction of the *R. davurica* root aqueous extract in the DSS-induced colitis model. UHPLC–MS/MS profiling resulted in tentative annotation of multiple constituents in SRP, offering preliminary chemical insights for subsequent investigation of its multicomponent pharmacological effects.

In this study, 5-ASA, a first-line clinical drug for UC, served as the positive control to evaluate the protective effects of SRP during DSS-induced acute colitis development in mice. Administration of the test fractions commenced concurrently with DSS exposure, allowing the experimental design to assess protective effects during disease induction rather than therapeutic efficacy after colitis establishment. Biological changes associated with SRP treatment were further explored by evaluating NF-*κ*B/NLRP3-associated inflammatory gene expression, intestinal epithelial barrier-associated proteins, fecal metabolic profiles, and gut microbiota composition.

## 2. Results

### 2.1. Tentative Chemical Profiling of SRP by UHPLC–Q Exactive Plus–MS/MS

**Figure 1:**
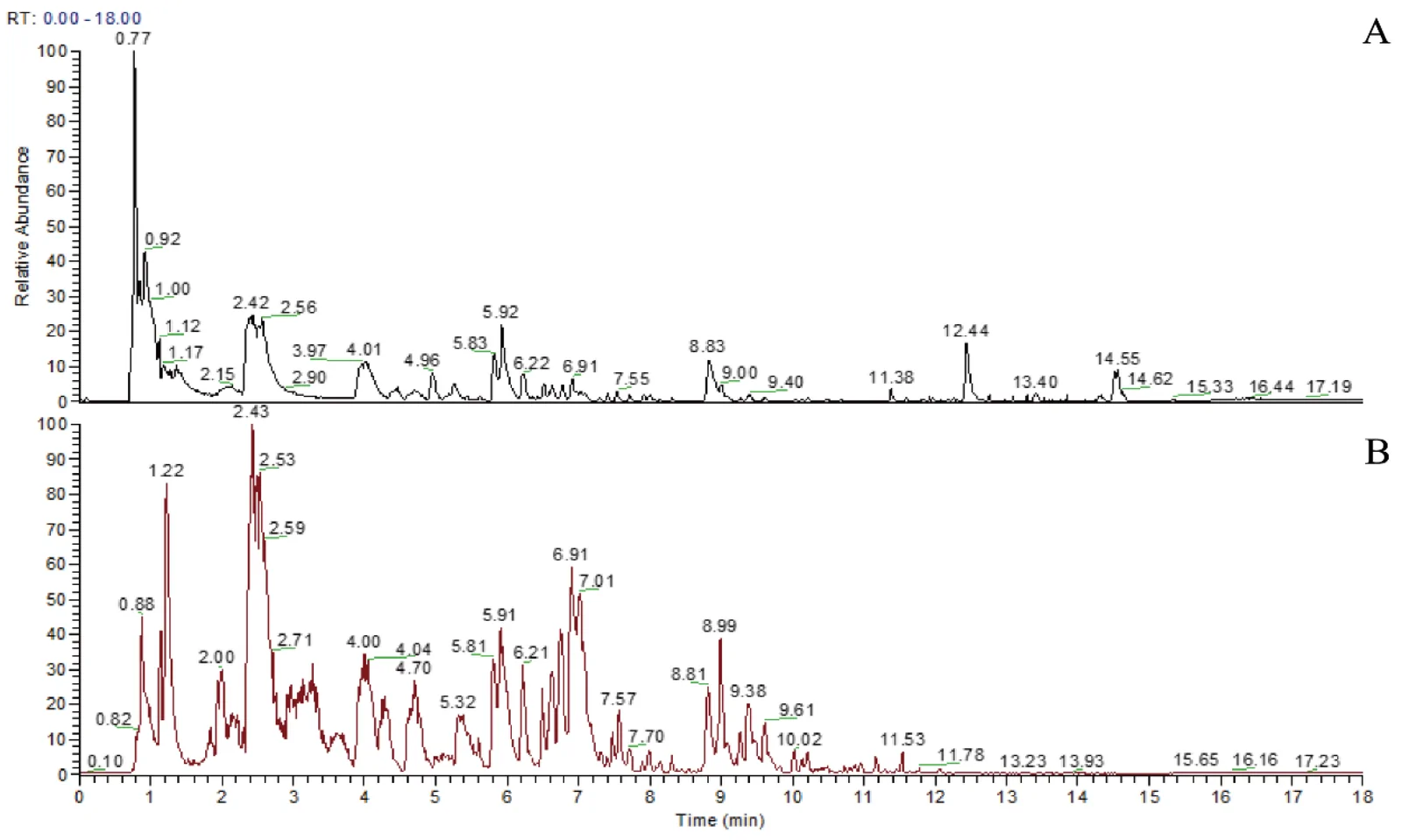
Base peak chromatograms of SRP from *Rosa davurica* Pall. root analyzed by UHPLC-Q Exactive Plus-MS/MS. (**A**) Base peak chromatogram acquired in positive ionization mode. (**B**) Base peak chromatogram acquired in negative ionization mode.

UHPLC–Q Exactive Plus–MS/MS profiling generated 47 and 21 mode-specific chromatographic features in the positive- and negative-ionization datasets, respectively. According to the identification-confidence criteria established in this study, all retained constituents were assigned Level 2 confidence. Due to the absence of authentic reference standards for compound confirmation, these annotations were not regarded as definitive identifications. Following cross-mode review and consolidation of duplicate annotations, a total of 68 nonredundant constituents were retained in the final chemical profile. These annotated constituents included several chemical classes such as flavonoids, phenolic acids and their derivatives, tannin-related compounds, fatty acid derivatives, lignans, phenylpropanoids, terpenoids, amino acids, nucleosides, and other small molecules. Notable polyphenol-related annotations included ellagic acid, kaempferol, naringenin, gallic acid, and caffeic acid. Table 1 and Table 2 summarize the 68 nonredundant annotations retained after cross-mode deduplication, while base peak chromatograms acquired in both ionization modes are displayed in Figure 1. Consequently, SRP should be considered a process-defined and qualitatively profiled fraction rather than a chemically standardized botanical preparation. Future studies will necessitate quantitative marker-based standardization.

Compounds in Table 1 and Table 2 were annotated at Level 2 confidence based on accurate mass, MS/MS fragmentation, retention behavior, and spectral/library matching, without authentic standard confirmation.

### 2.2. Effects of R. davurica Root Extracts and Fractions in DSS-Induced Colitis Mice

#### 2.2.1. Body Weight and DAI Score

**Figure 2:**
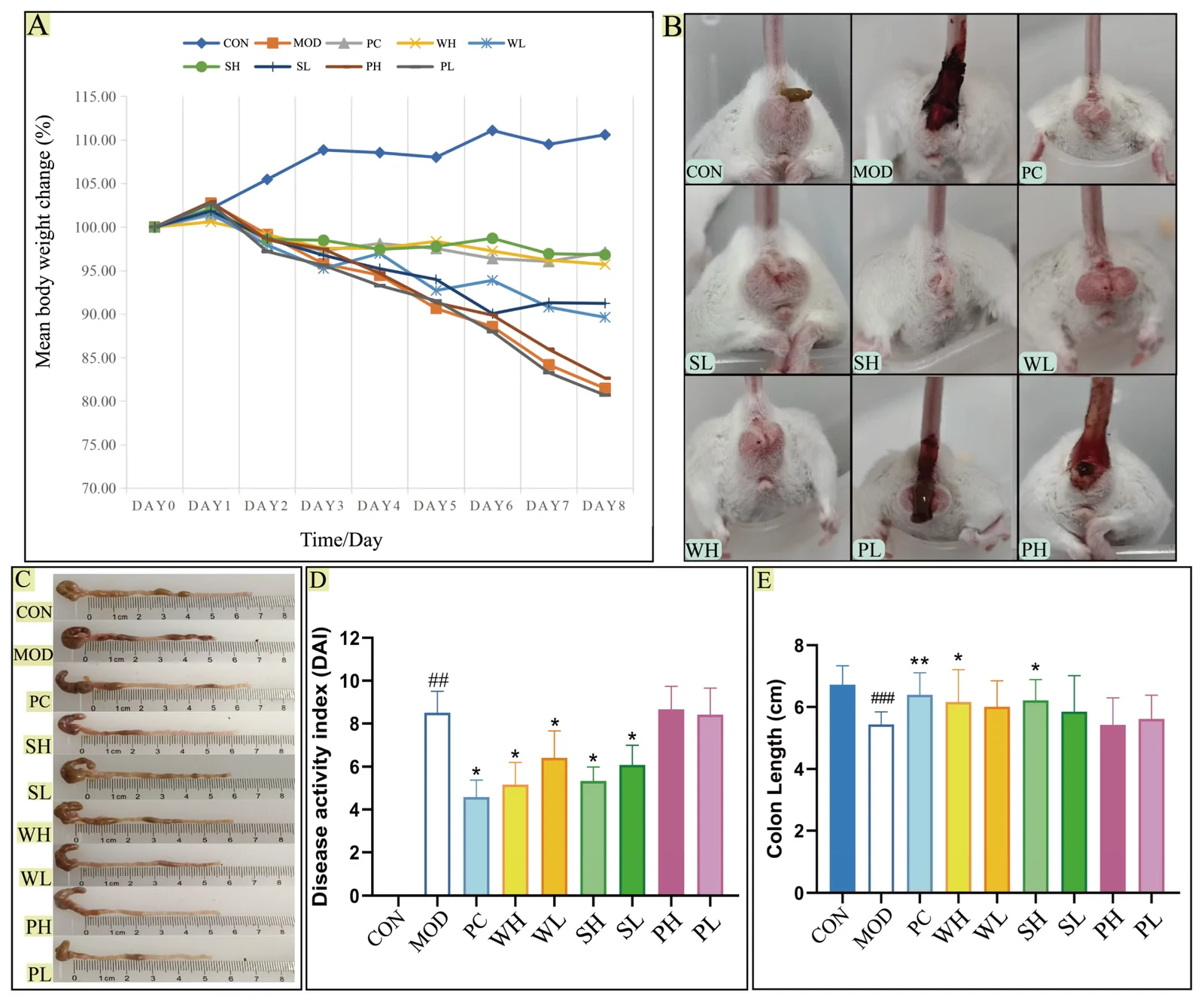
Effects of *R. davurica* root aqueous extract and its fractions on physiological parameters in DSS-induced acute colitis mice. (**A**) Body weight changes during the experimental period. (**B**) Representative photographs showing perianal bleeding and fecal characteristics. (**C**) Representative photographs of colon morphology and colon length. (**D**) DAI scores on day 8. (**E**) Colon length measured at necropsy. Data in (**A**,**D**,**E**) are presented as mean ± SD (*n* = 10 mice/group). * *p* < 0.05, ** *p* < 0.01 versus MOD group; ## *p* < 0.01, ### *p* < 0.001 versus CON group.

During the experiment, the CON group of mice exhibited a positive mental state, rapid response times, shiny and sleek fur, regular consumption of food and water, and a steady increase in body weight (Figure 2A). No obvious bleeding or swelling was observed around the anus in the CON group, and the feces maintained normal color, shape, and size. In contrast, significant abnormalities were noted in the MOD group, which displayed lethargy, a hunched posture, rough and dull fur, decreased food and water consumption, and marked weight loss. Additionally, swelling around the anal area, bleeding, and signs of inflammation were evident in the MOD group, along with loose stools and irregular stool shapes. Compared to the MOD group, the PC, WH, WL, SH, and SL groups demonstrated varying degrees of improvement in these conditions, with no evident peri-anal bleeding or swelling. Conversely, the PH and PL groups did not exhibit significant differences from the MOD group (Figure 2B).

Linear mixed-effects analysis of longitudinal body-weight changes revealed significant effects of treatment group (F(8, 81) = 24.428, *p* < 0.001), time (F(7, 567) = 146.120, *p* < 0.001), and the group × time interaction (F(56, 567) = 17.436, *p* < 0.001), indicating differences in body-weight trajectories among the experimental groups. Similarly, linear mixed-effects analysis of longitudinal DAI scores indicated significant effects of treatment group (F(8, 81) = 64.790, *p* < 0.001), time (F(7, 567) = 442.460, *p* < 0.001), and the group × time interaction (F(56, 567) = 12.247, *p* < 0.001), suggesting variation in the temporal progression of disease activity among the groups (Figure S5).

Relative to the CON group, the MOD group exhibited a significant increase in DAI score (*p* < 0.01). In comparison, DAI scores were significantly reduced in the PC, WH, WL, SH, and SL groups (all *p* < 0.01) relative to the MOD group, while no significant changes were observed in the PH and PL groups (Figure 2D). These results indicate that WRP and SRP provided significant protective effects against disease activity in UC mice under the present experimental conditions, whereas PRP did not demonstrate obvious protective effects.

#### 2.2.2. Colon Length

As presented in Figure 2C, the colon from the CON group of mice appeared normal, with an average length of 6.73 ± 0.61 cm. In contrast, the colons of MOD group mice exhibited edema, ulceration, and atrophy, resulting in an average length of only 5.43 ± 0.41 cm, which was significantly shorter than that of the CON group (*p* < 0.01). After administration, colonic appearance and length improved markedly in the PC, WH, and SH groups, yielding average lengths of 6.40 ± 0.71 cm, 6.16 ± 1.05 cm, and 6.22 ± 0.68 cm, respectively (all *p* < 0.05 or *p* < 0.01 compared to the MOD group). No significant changes in colon length were observed in the remaining groups (Figure 2E).

### 2.3. Histopathological Evaluation

**Figure 3:**
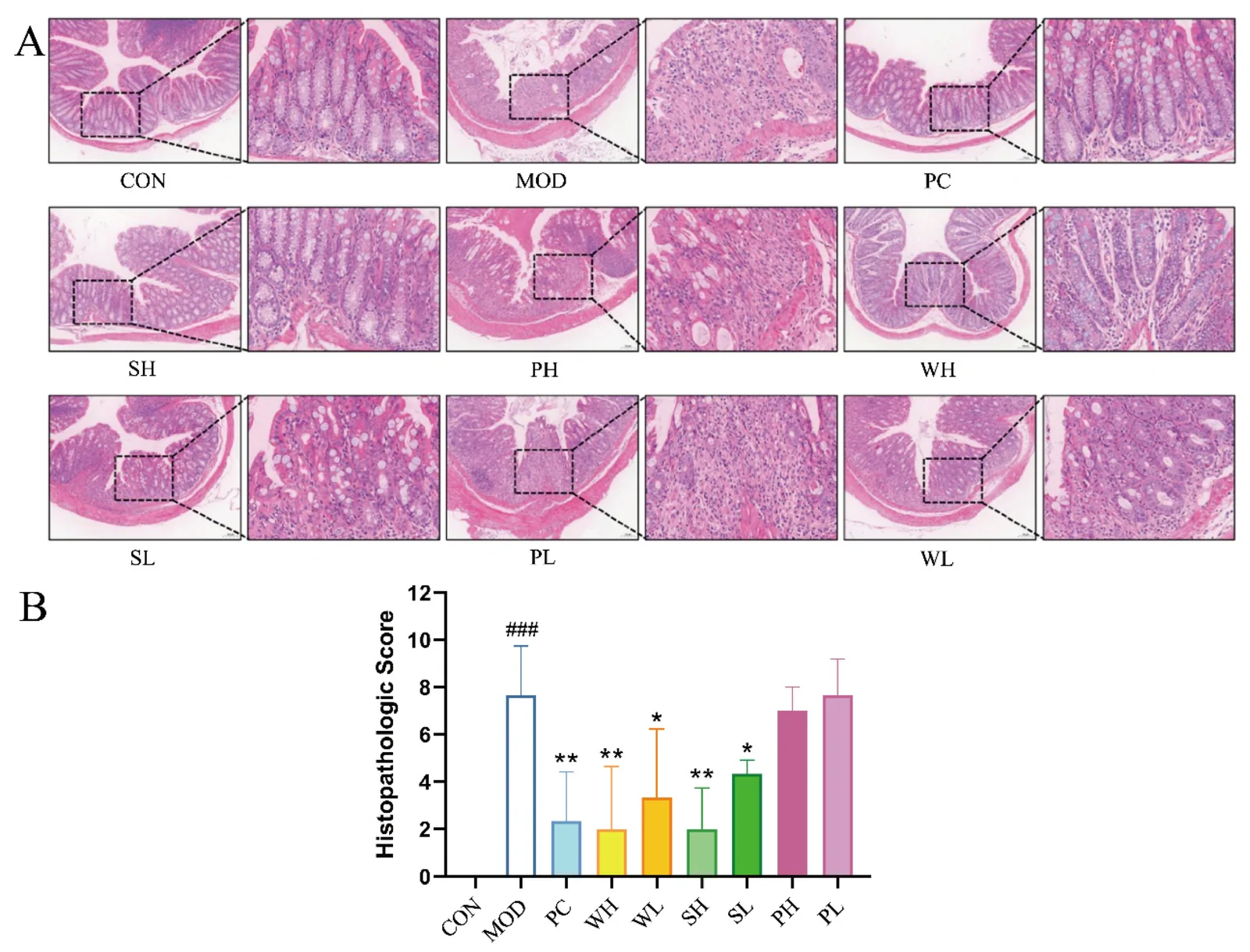
Histopathological evaluation of distal colon tissues from DSS-induced acute colitis mice. (**A**) Representative hematoxylin and eosin (HE)-stained sections of distal colon tissues at 100× and 400× magnifications. (**B**) Semi-quantitative histopathological scores. Data are presented as mean ± SD (*n* = 3 mice/group). * *p* < 0.05, ** *p* < 0.01 versus MOD group; ### *p* < 0.001 versus CON group.

Histological examination using hematoxylin and eosin (H&E) staining revealed that the colonic structure in the CON group was normal and intact, characterized by closely and regularly arranged epithelial cells, intact crypt architecture, and orderly intestinal glands. In contrast, the MOD group displayed severe disruption of these tissue structures due to DSS induction, resulting in extensive mucosal damage, ulceration, and massive inflammatory cell infiltration. As shown in Figure 3A, these pathologic abnormalities induced by DSS were substantially ameliorated following administration of WRP and SRP across various concentrations, along with the positive control drug 5-ASA.

All pathological features were quantified using a validated histopathological system, in which higher scores indicated more severe colonic tissue injury, and lower scores reflected greater histopathological protection [82]. Compared to the CON group, the MOD group exhibited a significantly elevated histopathological score (*p* < 0.001). In contrast, scores were significantly lower in the PC, WH, WL, SH, and SL groups compared to the MOD group (*p* < 0.05 or *p* < 0.01); however, the PH and PL groups did not show significant improvement over the MOD group (*p* > 0.05) (Figure 3B). These findings provide further support for the superior protective activity of SRP compared to PRP under the current experimental conditions.

### 2.4. Effects of SRP on the mRNA Expression of Inflammatory and Inflammasome-Associated Genes

**Figure 4:**
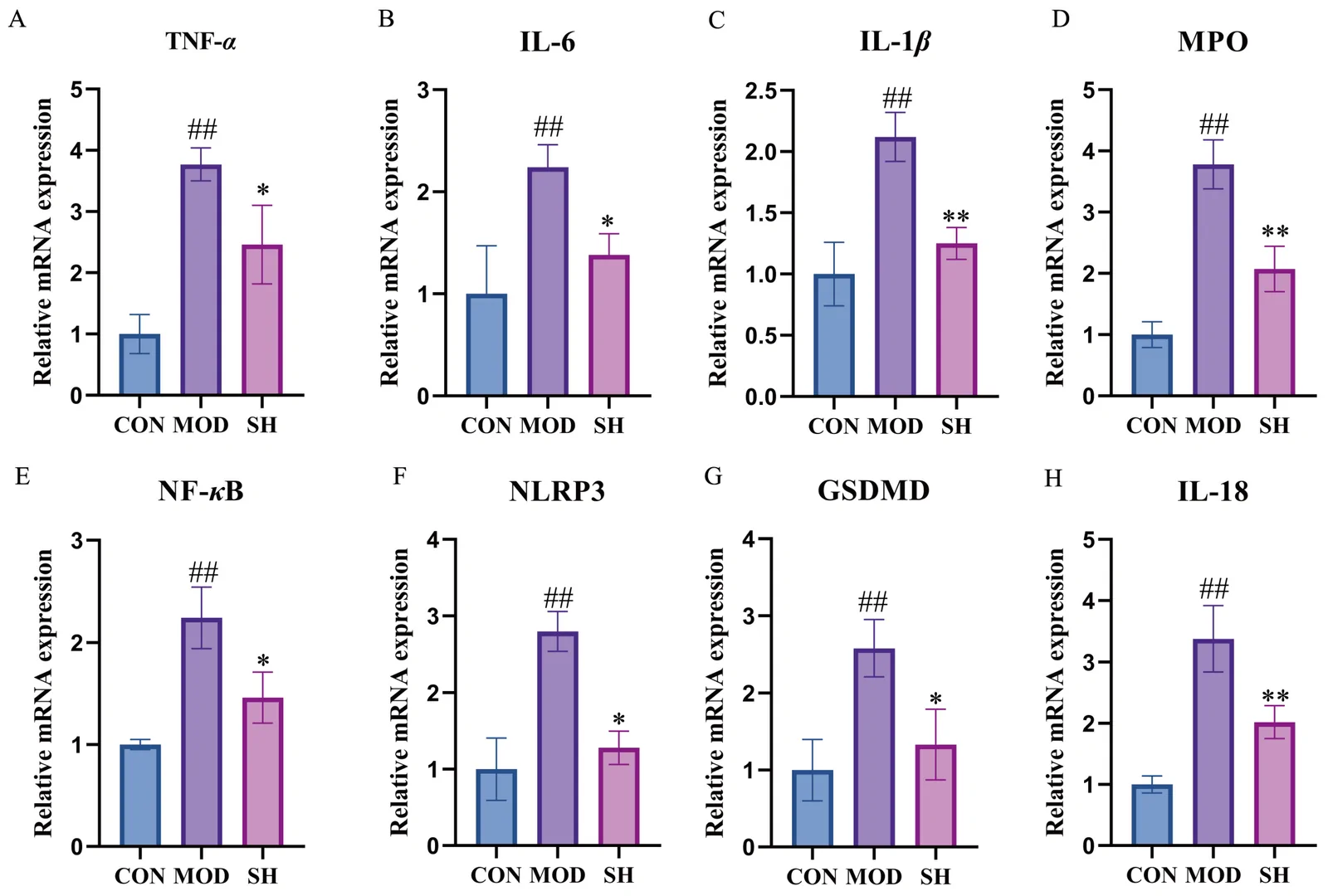
Relative mRNA expression of inflammatory and inflammasome-associated genes in colonic tissues of DSS-induced acute colitis mice. (**A**) TNF-*α.* (**B**) IL-6. (**C**) IL-1*β*. (**D**) MPO. (**E**) NF-*κ*B. (**F**) NLRP3. (**G**) GSDMD. (**H**) IL-18. mRNA expression levels were determined by RT-qPCR. Data are presented as mean ± SD (*n* = 3). * *p* < 0.05, ** *p* < 0.01 versus MOD group; ## *p* < 0.01 versus CON group.

Given that both the SH and SL groups significantly improved DAI scores and histopathological injury, while substantial amelioration of DSS-induced colon shortening was noted only in the SH group, the SH group was selected for RT-qPCR as the SRP treatment condition demonstrating a more consistent protective profile across the principal efficacy endpoints. In colon tissues, the MOD group showed significantly elevated mRNA levels of TNF-*α*, IL-6, IL-1*β*, MPO, NF-*κ*B, NLRP3, GSDMD, and IL-18 compared to the CON group (all *p* < 0.01). High-dose SRP significantly reduced the elevated mRNA levels of all eight genes compared to the MOD group (all *p* < 0.05). The NF-κB mRNA level in the SH group did not differ significantly from that of the CON group (*p* > 0.05) (Figure 4). These RT-qPCR findings indicate transcriptional changes in inflammatory and inflammasome-associated genes; however, they do not confirm changes in protein abundance, NF-*κ*B phosphorylation or nuclear translocation, NLRP3 inflammasome assembly, or downstream protein cleavage.

### 2.5. Effects of SRP on Barrier-Associated Protein Expression

**Figure 5:**
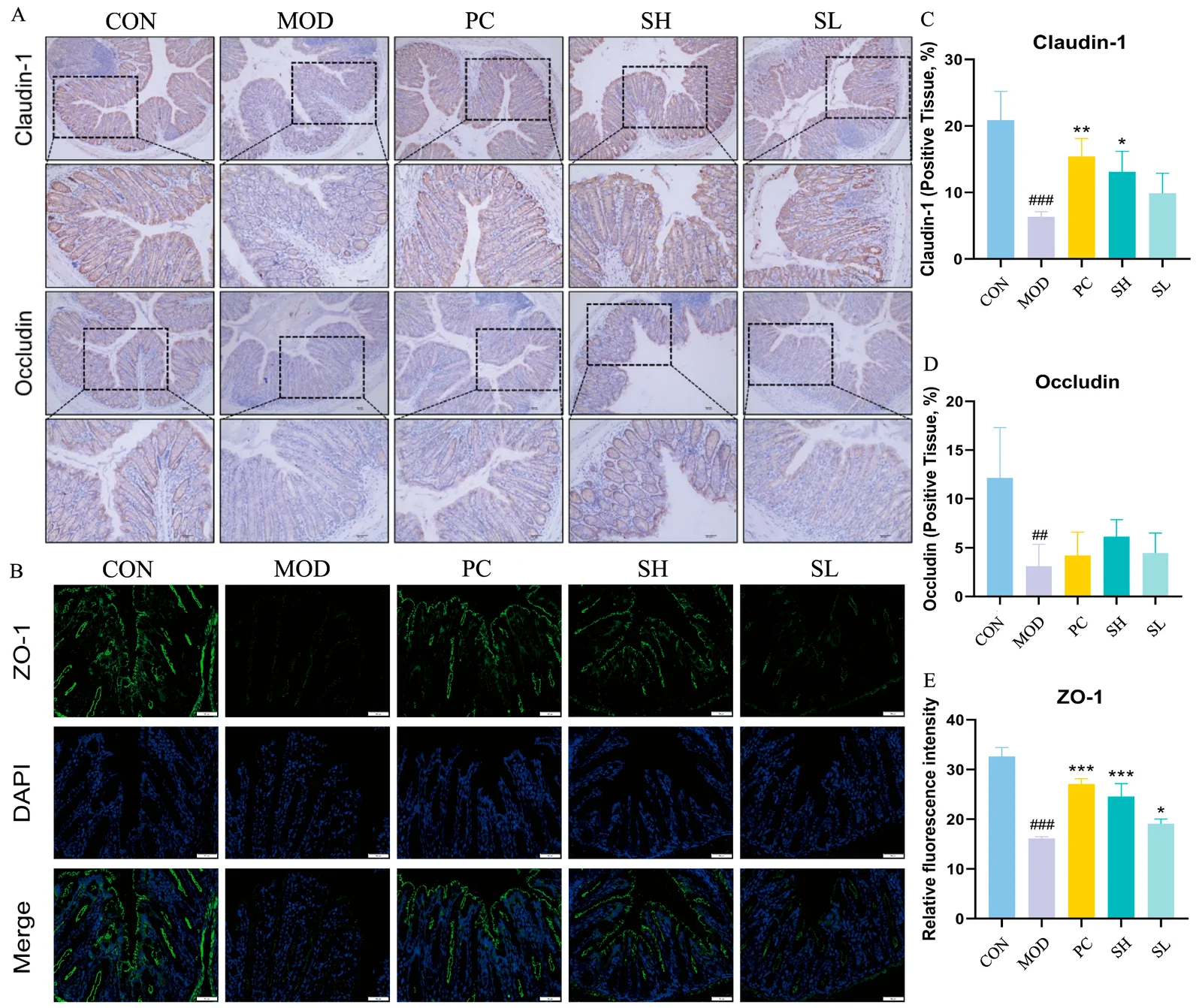
Expression of tight junction proteins in colonic epithelial tissues assessed by immunohistochemistry and immunofluorescence. (**A**) Representative immunohistochemical images of Claudin-1 and Occludin staining. Dashed boxes indicate enlarged regions. (**B**) Representative immunofluorescence images showing ZO-1 staining (green), DAPI nuclear staining (blue), and merged images. (**C**–**E**) Quantitative analysis of Claudin-1, Occludin, and ZO-1 expression. Claudin-1 and Occludin levels were quantified as the percentage of DAB-positive tissue area, and ZO-1 expression was quantified as relative mean fluorescence intensity. Data are presented as mean ± SD (*n* = 3 mice/group randomly selected from the original cohort). * *p* < 0.05, ** *p* < 0.01, *** *p* < 0.001 versus MOD group; ## *p* < 0.01, ### *p* < 0.001 versus CON group. bar = 50.

Immunohistochemical and immunofluorescence staining revealed that SRP treatment modulated the expression and distribution of intestinal barrier-associated proteins in colonic tissues (Figure 5). Compared to the CON group, the MOD group exhibited significantly decreased expression of Claudin-1 and Occludin (*p* < 0.001 and *p* < 0.01, respectively). Claudin-1 expression was significantly higher in the PC and SH groups than in the MOD group (both *p* < 0.05); however, Occludin expression was not significantly altered in either the PC or SH group compared with the MOD group (*p* > 0.05). The SL group showed no significant changes in Claudin-1 or Occludin expression compared to the MOD group (*p* > 0.05) (Figure 5C,D). ZO-1 expression was significantly decreased in the MOD group compared to the CON group (*p* < 0.01), while the PC, SH, and SL groups exhibited significantly increased ZO-1 expression relative to the MOD group (PC, SH vs MOD *p* < 0.001; SL vs MOD *p* < 0.05) (Figure 5E). Collectively, SRP treatment produced differential changes among the assessed barrier-associated proteins: high-dose SRP enhanced Claudin-1 expression, both doses of SRP increased ZO-1 expression, whereas neither dose significantly restored Occludin expression.

### 2.6. SRP-Associated Changes in Fecal Metabolic Profiles in DSS-Induced Colitis

**Figure 6:**
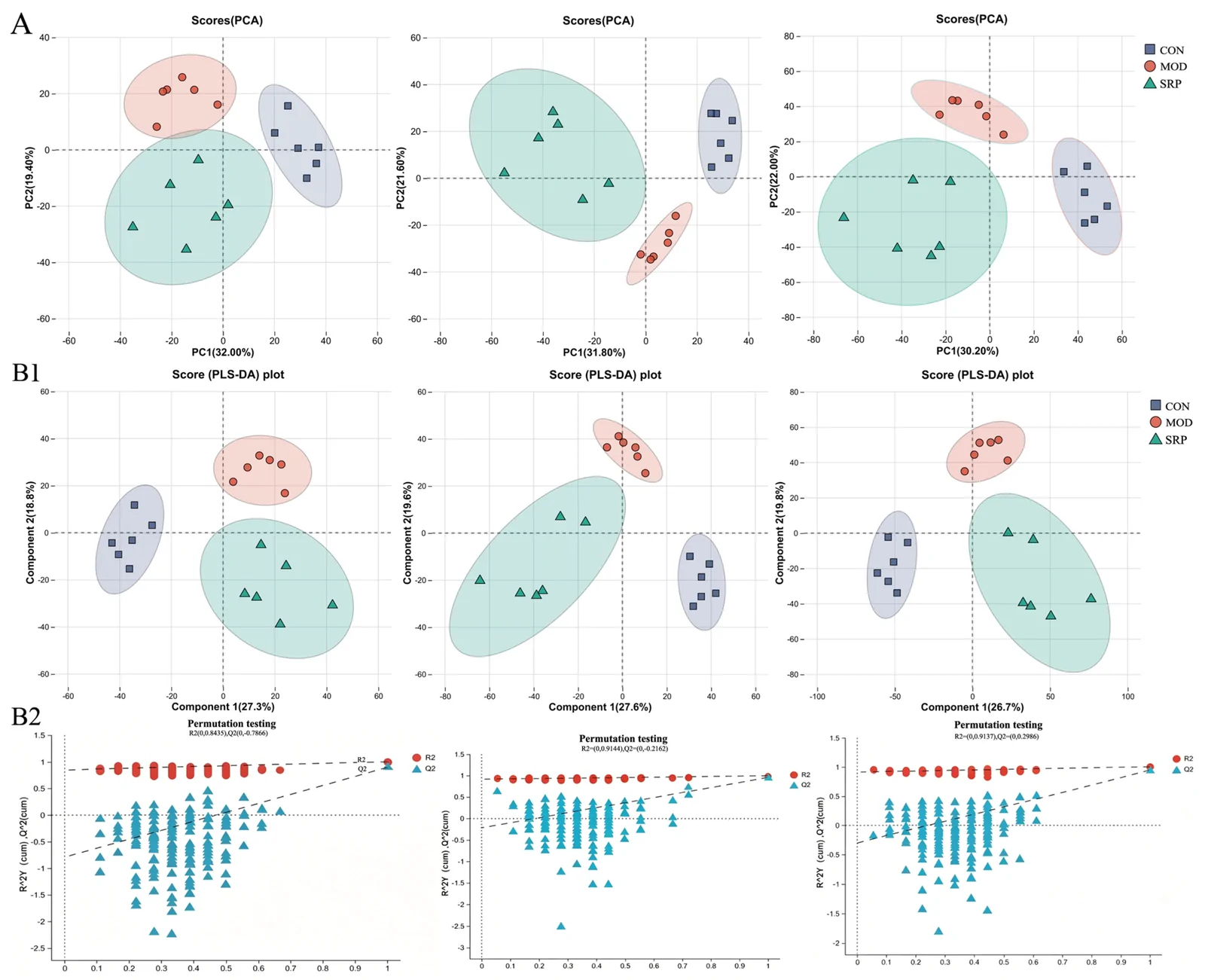
Multivariate analysis of untargeted fecal metabolomics data. (**A**) PCA score plots of the POS, NEG, and combined datasets. Percentages in parentheses indicate the variance explained by PC1 and PC2. (**B1**) PLS-DA score plots of the corresponding datasets. (**B2**) Permutation-validation plots for the PLS-DA models. Each symbol represents an individual biological sample (*n* = 6 mice/group).

PCA showed distinct separation of fecal metabolic profiles among the CON, MOD, and SRP groups in the POS, NEG, and combined datasets (Figure 6A). PLS-DA also indicated separation among the three groups, and permutation testing yielded Q^2^ intercepts below zero across all datasets, suggesting no apparent model overfitting (Figure 6(B1,B2)).

**Figure 7:**
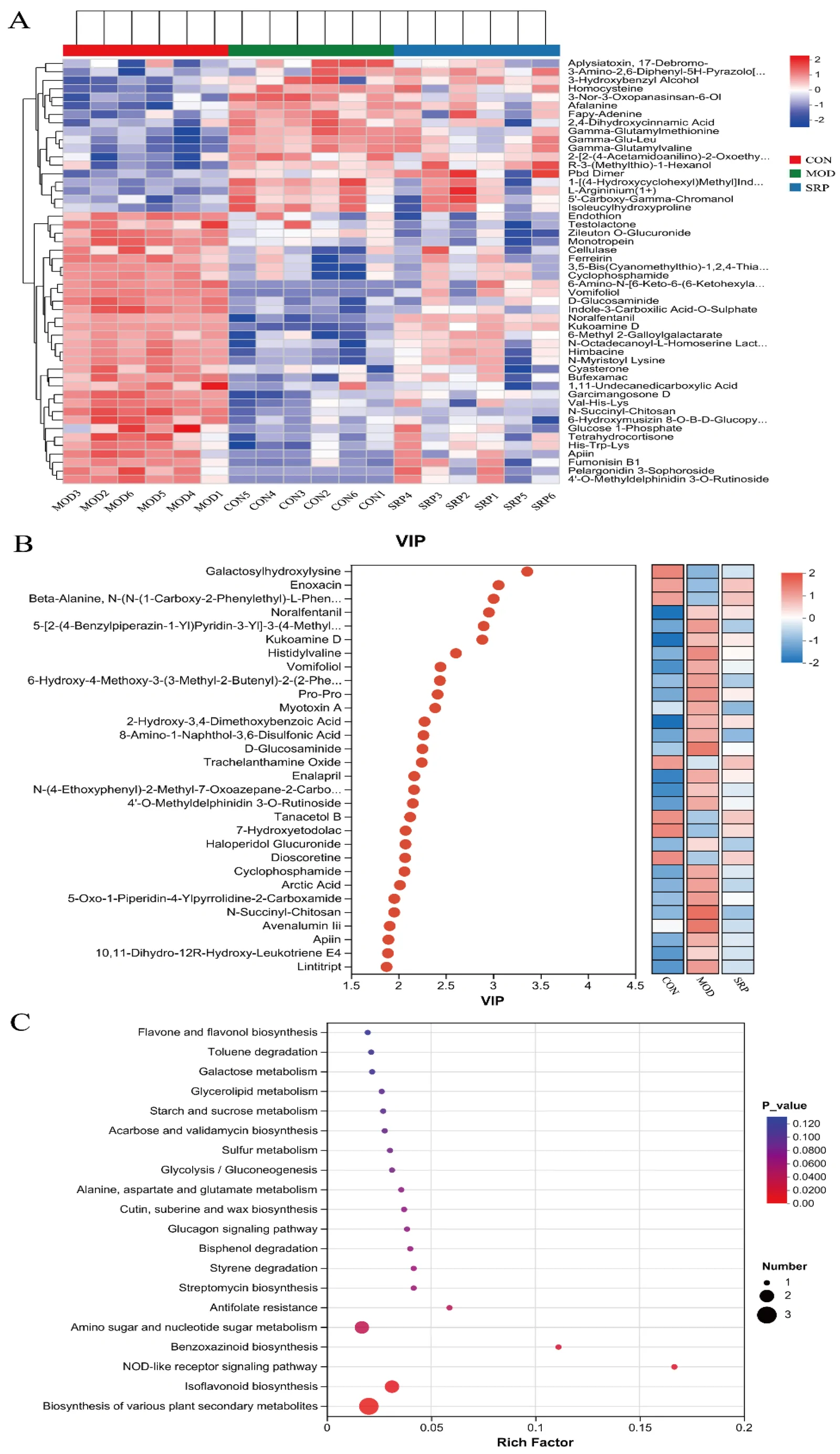
Candidate fecal metabolite profiling and pathway enrichment analysis. (**A**) Hierarchical clustering heatmap of candidate metabolites meeting the screening criteria of VIP > 1 and nominal *p* < 0.05 among the CON, MOD, and SRP groups. The color scale represents z-score-normalized relative abundance (red, higher abundance; blue, lower abundance). (**B**) VIP score plot (left) and fold-change heatmap (right) of candidate metabolites. (**C**) KEGG pathway enrichment analysis of candidate metabolites. Bubble size indicates the number of enriched metabolites, and color intensity indicates enrichment significance.

Candidate metabolites were screened based on VIP > 1 and a nominal *p* < 0.05. Hierarchical clustering analysis revealed metabolite abundance patterns dependent on group, with SRP-treated samples partially separating from the MOD group (Figure 7A). Several candidate metabolites meeting the screening criteria showed group-dependent abundance patterns. Notably, both 10,11-dihydro-12R-hydroxy-leukotriene E4 and D-glucosaminide exhibited higher relative abundance in the MOD group than in the CON group and showed a reduction following SRP treatment (Figure 7B). FDR-adjusted q values for the candidate metabolites are provided in Table S2. Additional variations were observed among metabolites related to amino acids and lipids. As these metabolites were putatively annotated in an untargeted analysis, their identities and quantitative changes require targeted confirmation. Detailed statistical information for representative candidate metabolites is provided in Table S2.

KEGG enrichment analysis linked the candidate metabolites to pathways such as NOD-like receptor signaling and amino sugar and nucleotide sugar metabolism (Figure 7C). These enrichment results suggest that the altered fecal metabolites were statistically associated with annotations related to inflammation- and carbohydrate metabolism; however, they do not confirm functional regulation of these pathways by SRP.

### 2.7. SRP-Associated Changes in Gut Microbiota Composition in DSS-Induced Colitis Mice

**Figure 8:**
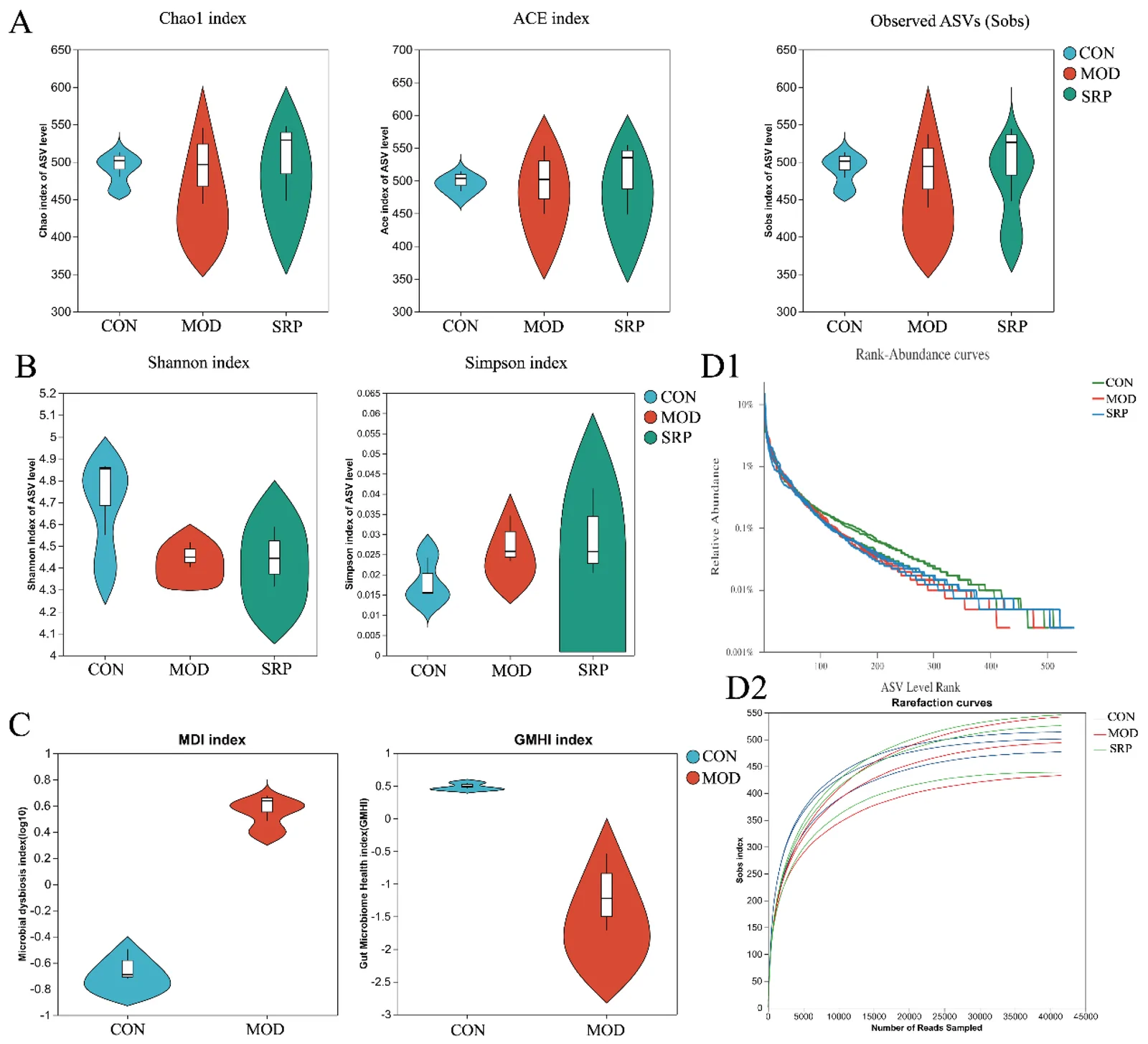
*α*-diversity analysis and sequencing depth evaluation of gut microbiota in DSS-induced colitis mice. (**A**) Chao1, ACE, and observed ASV (Sobs) indices. (**B**) Shannon and Simpson indices. (**C**) MDI and GMHI indices comparing the CON and MOD groups. (**D1**) Rank-abundance curves showing species richness and evenness. (**D2**) Rarefaction curves based on the Sobs index. Data in (**A**–**C**) are presented as violin plots overlaid with box plots (*n* = 3 mice/group randomly selected from the original cohort).

The 16S rRNA gene sequencing results indicated that rarefaction curves approached saturation and Good’s coverage values exceeded 0.999 across all groups (Figure 8(D2)), demonstrating adequate sequencing coverage. No significant differences were observed in the Sobs, ACE, Chao1, Shannon, or Simpson indices among the CON, MOD, and SRP groups (Figure 8A,B).

**Figure 9:**
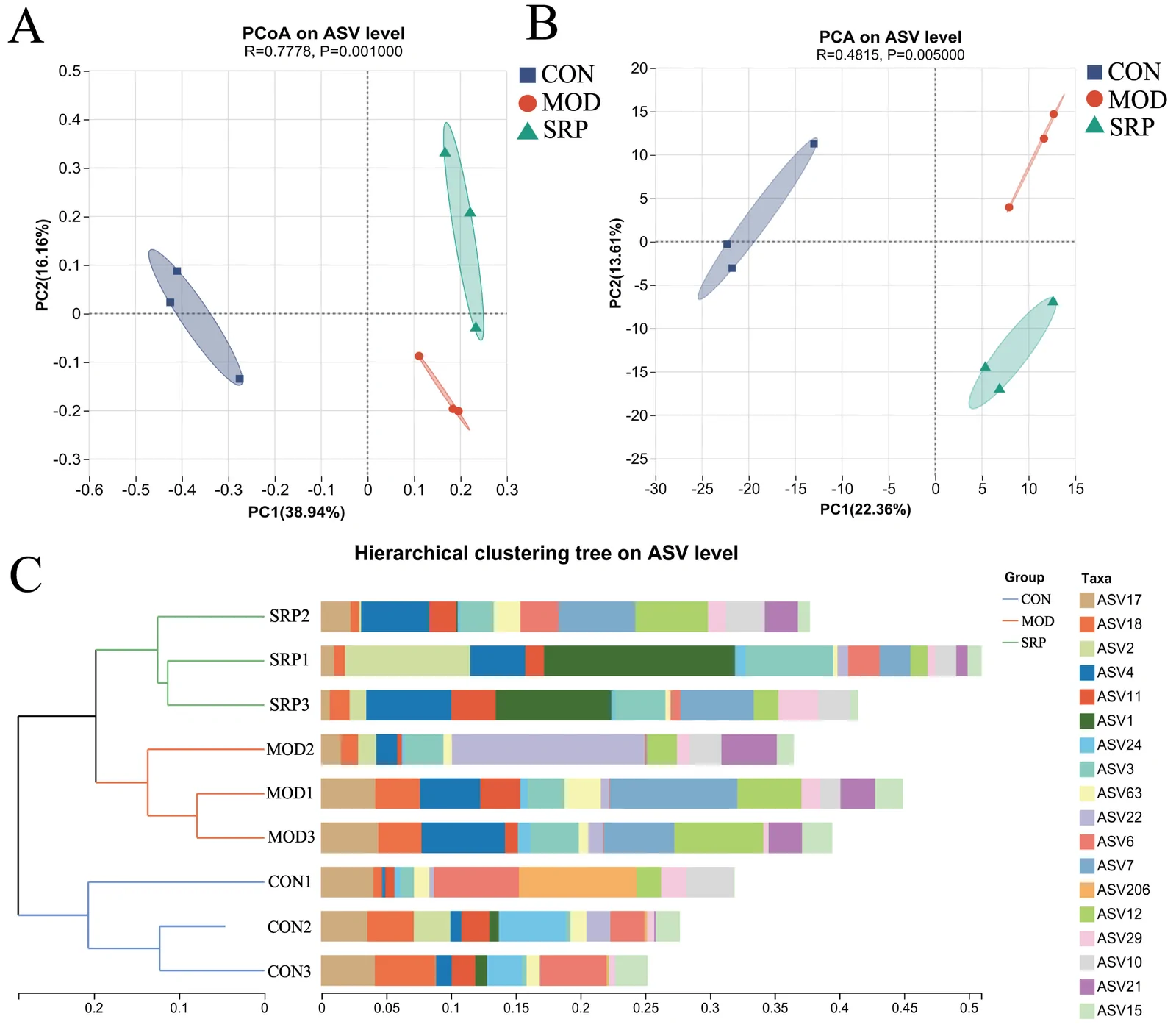
*β*-diversity analysis of gut microbiota at the ASV level. (**A**) PCoA and (**B**) PCA score plots based on ASV-level microbial profiles. Each symbol represents an individual biological sample (*n* = 3 mice/group randomly selected from the original cohort). (**C**) Hierarchical clustering tree combined with stacked bar plots showing the relative abundance of dominant ASVs. PERMANOVA results were as follows: PCA, R = 0.4815, *p* = 0.005; PCoA, R = 0.7778, *p* = 0.001.

PCA and PCoA analyses revealed distinct separation among the three groups (Figure 9A,B), reflecting differences in microbial community structure. Hierarchical clustering analysis further supported these alterations, showing group-dependent clustering patterns (Figure 9C).

**Figure 10:**
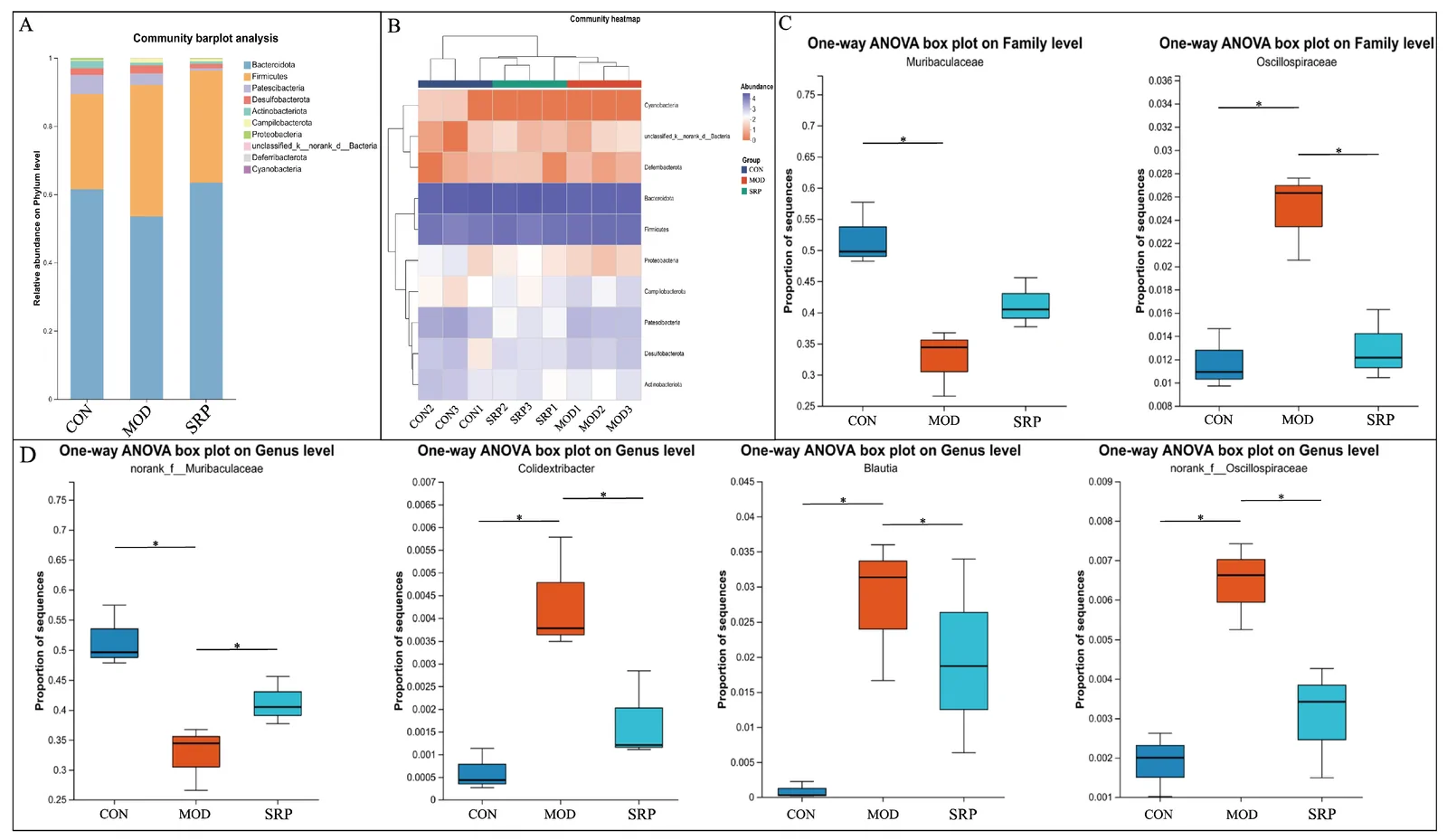
Gut microbiota composition and group-associated taxonomic differences at different taxonomic levels. (**A**) Relative abundance of bacterial taxa at the phylum level among the CON, MOD, and SRP groups. (**B**) Phylum-level heatmap with hierarchical clustering of samples. (**C**) Relative abundance of differential bacterial families including *Muribaculaceae* and *Oscillospiraceae*. (**D**) Relative abundance of differential genera including *norank_f_Muribaculaceae*, *Colidextribacter*, *Blautia*, and *norank_f_Oscillospiraceae*. Differential taxa were identified by LEfSe analysis (LDA score > 4.0, *p* < 0.05). Data in (**C**,**D**) are presented as box plots (*n* = 3 mice/group). * *p* < 0.05 for indicated comparisons.

At the phylum level, *Bacteroidota* and *Firmicutes* were predominant across all groups. UC modeling altered the relative abundance distribution of major bacterial phyla, while SRP treatment was linked to partial shifts in these relative abundance patterns (Figure 10A,B). At more granular taxonomic levels, community composition analysis revealed differences in several dominant taxa among the groups. Notably, at the genus level, *norank_f_Muribaculaceae* showed lower relative abundance in the MOD group and increased following SRP treatment. Conversely, *Colidextribacter* and *Blautia* exhibited increased levels after DSS induction, which subsequently decreased after SRP treatment. *norank_f_Oscillospiraceae* similarly showed higher relative abundance in the MOD group and lower relative abundance following SRP treatment. (Figure 10C,D).

**Figure 11:**
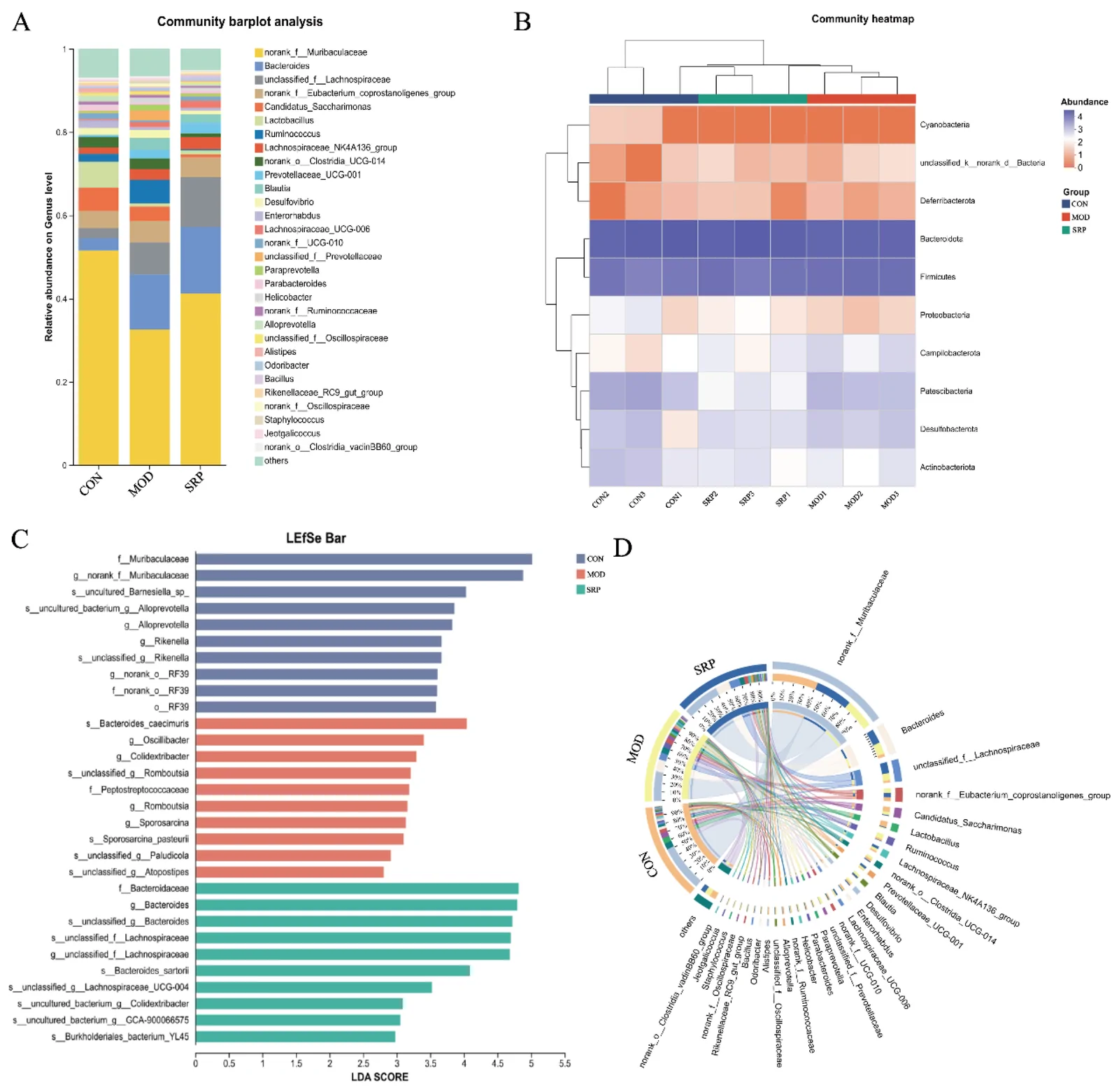
Gut microbiota community composition and LEfSe-selected taxonomic features. (**A**) Relative abundance of dominant bacterial genera among the CON, MOD, and SRP groups. (**B**) Genus-level heatmap with hierarchical clustering of samples. (**C**) LEfSe analysis showing taxonomic features meeting the specified LDA and p-value criteria from phylum to genus levels (LDA score > 4.0, *p* < 0.05). (**D**) Circos plot illustrating associations between experimental groups and genus-level bacterial taxa. The microbiota analyses were based on *n* = 3 mice/group.

LEfSe analysis identified group-associated taxonomic features, including *Bacteroidaceae* and *Bacteroides* in the SRP group (LDA > 4.0, *p* < 0.05) (Figure 11C). Heatmap and Circos analyses consistently demonstrated group-specific microbial distribution patterns (Figure 11A,B,D).

Overall, SRP intervention did not significantly alter microbial *α*-diversity but was associated with differences in microbial community structure and the relative abundance of several taxa compared with the MOD group.

## 3. Discussion

The present study provides pharmacological evidence supporting SRP as a potentially important bioactive fraction derived from the aqueous extract of *R. davurica* root and demonstrates its protective activity in DSS-induced acute colitis mice. Since SRP administration was initiated concurrently with DSS exposure, these findings reflect protective effects during the development of DSS-induced colitis rather than therapeutic reversal of established disease. Compared to PRP, SRP exhibited stronger protective effects, including alleviation of disease severity, reduction in histological injury, and improvement in inflammatory responses (Figure 2 and Figure 3). These findings suggest that the anti-colitis activity of *R. davurica* root aqueous extract is primarily associated with the SRP rather than the crude polysaccharide precipitate. Notably, the principal in vivo efficacy outcomes, such as body weight, DAI, and colon length, were evaluated in the full cohort of 10 mice per group, while histopathological analysis was conducted as a downstream tissue-level assessment in a randomly selected subset of three mice per group. Thus, histological analysis was intended to provide complementary morphological evidence supporting the full cohort efficacy findings rather than serving as the sole basis for evaluating treatment efficacy. To minimize potential selection and observer bias, animals used for histopathology were selected without reference to disease severity, colon length, or subsequent experimental outcomes. Histological scoring was independently performed by two investigators who were blinded to group allocation.

The qualitative chemical profile offers preliminary context for interpreting the pharmacological activity of SRP. Among the tentatively annotated constituents, ellagic acid, kaempferol, naringenin, gallic acid, and caffeic acid have previously been investigated in experimental models of intestinal inflammation. Ellagic acid has been reported to alleviate DSS-induced colitis and reduce inflammatory and histopathological injury in both acute and chronic experimental contexts [83,84]. Kaempferol has also demonstrated protective effects in DSS-induced colitis, including attenuation of inflammatory responses and preservation of intestinal barrier-associated proteins [85]. In separate studies, naringenin has been shown to promote recovery from DSS-induced colonic injury and was associated with reduced inflammatory responses and changes in epithelial junction-associated proteins [86]. Similarly, both gallic acid and caffeic acid have been reported to mitigate disease manifestations and inflammatory responses in DSS-induced colitis models [87,88]. These prior findings provide a plausible pharmacological context for the presence of these constituents in SRP and align directionally with some of the inflammatory and barrier-associated changes observed in the current study. However, the detection of a constituent in SRP and its previously reported activity as an isolated compound do not confirm that it contributed materially to the observed effects. Moreover, none of the individual constituents were pharmacologically evaluated in this study. Consequently, these compounds should be regarded as representative and potentially relevant constituents rather than established active principles. The protective activity of SRP may reflect the collective effects of multiple constituents, though additive, synergistic, or antagonistic interactions were not examined. Further quantitative chemical characterization and bioactivity-guided studies will be necessary to identify the constituents that significantly contribute to the activity of SRP. Once quantitatively predominant or bioactivity-relevant constituents and experimentally relevant mucosal protein targets are established, structure-based in silico analyses may facilitate the prioritization of plausible compound–protein interactions for subsequent experimental validation. However, due to the qualitative nature of the present UHPLC–MS/MS analysis, which did not establish either major active constituents or direct protein targets, such analyses were not performed in the current study.

Inflammatory dysregulation is a central feature of UC, with excessive activation of NF-*κ*B and NLRP3 inflammasome signaling contributing to cytokine production, immune amplification, and intestinal tissue damage. Prior studies have indicated that natural products can mitigate experimental colitis by modulating NF-*κ*B-associated inflammatory responses and NLRP3 inflammasome activation [89,90,91]. Additionally, NF-*κ*B-mediated priming represents a critical step preceding NLRP3 inflammasome activation [92]. In the present study, SRP treatment diminished DSS-induced increases in the mRNA expression levels of NF-*κ*B, NLRP3, GSDMD, IL-18, TNF-*α*, IL-1*β*, IL-6, and MPO (Figure 4). Although both SRP doses significantly improved DAI scores and histopathological injury, the high-dose group demonstrated a more consistent protective profile across key in vivo efficacy endpoints, including significant alleviation of DSS-induced colon shortening. Consequently, the high-dose SRP group was selected for RT-qPCR analysis, with resulting transcriptional data interpreted to characterize this treatment condition rather than to establish a dose-dependent molecular response. These transcriptional alterations, co-occurring with reduced inflammatory and histopathological injury, reflect SRP-associated modulation of inflammatory and inflammasome-associated gene expression. However, caution is warranted in interpreting these findings, as the current evidence primarily relies on transcriptional changes. Further validation at the protein and functional levels is necessary to determine whether SRP directly influences NF-*κ*B activation, NLRP3 inflammasome assembly, pyroptosis-related protein expression, or upstream inflammatory events. Given that SRP is a fraction derived from a medicinal plant rather than a single purified compound, its anti-inflammatory effects may result from the synergistic actions of multiple bioactive constituents. Previous studies have reported that bioactive fractions from plants can exert intestinal protective effects accompanied by changes in inflammatory responses, oxidative balance, epithelial barrier function, and the intestinal microenvironment [93,94]. Thus, the observed reduction in inflammatory gene expression should not be construed as evidence of SRP directly targeting a singular signaling pathway. Due to the complex composition of SRP, these transcriptional changes may result from various direct or indirect processes, including alterations in the intestinal inflammatory environment.

In addition to the inflammatory transcriptional changes, SRP treatment was associated with differential changes among the assessed barrier-associated proteins. High-dose SRP enhanced Claudin-1 expression, whereas both SRP doses elevated ZO-1 expression; however, Occludin expression did not show significant recovery. Notably, Occludin also remained unchanged following 5-ASA treatment, suggesting that the limited response of Occludin was not exclusive to SRP under the current experimental conditions. This differential pattern may reflect protein-specific responses, varying rates of turnover or recovery among tight-junction components, or differences in the temporal sequence of junction restoration after DSS-induced injury. As only a single experimental time point was evaluated, these possibilities remain unexamined and should be viewed as hypotheses rather than confirmed mechanisms. Claudin-1 and Occludin quantification utilized the same immunohistochemical approach, whereas ZO-1 was assessed through immunofluorescence. Consequently, direct comparisons of the magnitude of ZO-1’s response with those of Claudin-1 or Occludin are inappropriate. Moreover, the differing responses of Claudin-1 and Occludin cannot be solely attributed to variations in detection methodologies, as both proteins were assessed using similar analytical techniques.

Inflammatory injury and epithelial barrier disruption are closely interconnected in colitis, with reduced inflammatory and histopathological injury potentially facilitating the recovery of selected junction-associated proteins [95]. The observed increases in Claudin-1 and ZO-1 may result from a direct effect of SRP on epithelial cells, a secondary consequence of diminished inflammatory injury, or a combination of both processes. These explanations are not mutually exclusive, and the present study cannot differentiate among them. Additionally, due to the absence of an isolated epithelial model or measurements of functional intestinal permeability, a direct epithelial-protective effect of SRP cannot be established. While the observed changes suggest partial improvement in selected barrier-associated proteins, they do not confirm functional restoration of intestinal permeability. The evaluation of additional junction-associated proteins and functional permeability assays would provide a more comprehensive assessment. Thus, the barrier-related findings should be interpreted as treatment-associated changes in protein expression rather than as evidence of direct or functional restoration of the intestinal barrier. Untargeted fecal metabolomics yielded complementary evidence of SRP-associated changes in fecal metabolic composition. Multivariate analysis revealed distinct metabolic profiles among the CON, MOD, and SRP groups, indicating that DSS induction and SRP treatment were linked to alterations in fecal metabolites (Figure 6 and Figure 7). Previous studies have shown that fecal metabolic changes are closely associated with the progression of inflammatory bowel disease and may reflect the combined effects of host metabolism, microbial activity, and shifts in the intestinal environment [96]. Among the candidate metabolic features identified through the exploratory screening, 10,11-dihydro-12R-hydroxy-leukotriene E4 was tentatively annotated and displayed a higher relative abundance in the MOD group, with a shift toward the CON-group pattern following SRP treatment. This compound has been recognized as a downstream leukotriene-related metabolite. Leukotrienes, derived from arachidonic acid, are inflammatory lipid mediators, and elevated leukotriene production has been reported in active UC and experimental colitis [97]. Additionally, urinary leukotriene E4 has been investigated as a potential indicator of inflammatory activity in IBD [98]. The observed abundance pattern therefore provides exploratory context for leukotriene-related inflammatory metabolism. However, since the metabolite identity is based on untargeted annotation and leukotriene synthesis was not measured directly, the current data do not confirm that SRP inhibited leukotriene production or signaling. D-Glucosaminide was also tentatively annotated and showed a similar increase in the MOD group and decrease in the SRP group. This change may provide context for the enrichment of amino sugar and nucleotide sugar metabolism, pathways associated with intestinal carbohydrate processing, microbial substrate utilization, and glycoconjugate biosynthesis. Colonic mucins are extensively glycosylated, and disruption of mucin O-glycans has been linked to impaired mucus-barrier integrity and increased vulnerability to colitis [99]. Thus, the enrichment of amino sugar and nucleotide sugar metabolism may reflect changes in the intestinal luminal and mucosal metabolic environment during DSS-induced injury and SRP treatment. Nevertheless, the abundance of fecal D-glucosaminide does not directly measure mucin glycosylation, glycosyltransferase activity, or epithelial barrier function; therefore, this interpretation should be viewed in context and as hypothesis-generating rather than mechanistic. KEGG enrichment analysis further mapped the candidate metabolites to pathways, including NOD-like receptor signaling and amino sugar and nucleotide sugar metabolism (Figure 7). These metabolic patterns align with the reduced inflammatory gene expression and histopathological injury observed after SRP treatment. However, pathway enrichment does not demonstrate functional regulation of NOD-like receptor signaling nor establish that the identified metabolites mediated the protective effects of SRP. Accordingly, the reported metabolic features should be regarded as exploratory candidates rather than validated biomarkers or definitive disease-associated metabolites. Given the limited number of biological replicates, the metabolomic findings should be interpreted as exploratory and associative evidence, requiring validation in larger independent cohorts and targeted studies to clarify their biological relevance.

Gut microbiota analysis further revealed SRP-associated changes in the microbial community of DSS-treated mice. Although SRP did not significantly affect *α*-diversity indices, *β*-diversity analysis indicated clear separation among the CON, MOD, and SRP groups, suggesting that SRP treatment was associated with differences in microbial community structure rather than overall microbial richness (Figure 8 and Figure 9). At the taxonomic level, DSS induction and SRP treatment were associated with differential abundance patterns of several taxa, including *norank_f_Muribaculaceae*, *Blautia*, *Colidextribacter*, and *norank_f_Oscillospiraceae* (Figure 10 and Figure 11). Of particular interest is *Muribaculaceae*, as members of this bacterial family are associated with the utilization of complex carbohydrates and the fermentation of dietary or host-derived substrates. Alterations in the abundance of *Muribaculaceae* have also been observed in experimental colitis, suggesting that changes within this family may mirror disturbances in the intestinal microbial and nutritional environment. In the present study, the SRP-associated change in *norank_f_Muribaculaceae* was directionally consistent with partial shifts in the composition of the DSS-disturbed/associated microbial community [100]. However, because microbial carbohydrate utilization and fermentation products were not directly measured, the current data do not establish a functional role for *Muribaculaceae* in the protective effects of SRP. Marked treatment-associated changes were also observed in *Blautia* and *Colidextribacter*. Previous studies have linked *Blautia* to intestinal metabolic homeostasis and short-chain fatty acid-related functions. Nonetheless, its relationship with intestinal inflammation varies among species, strains, host conditions, and disease contexts. Thus, the observed change in *Blautia* should not be interpreted as a uniform classification of the genus as either beneficial or detrimental. Similarly, *Colidextribacter* has been reported to change in response to experimental intestinal inflammation, but its biological significance lacks consistency across different intervention models [101,102,103]. Therefore, SRP-associated changes in these genera are best viewed as components of broader changes in microbial community composition, rather than as evidence that modulation of any single bacterial taxon mediated the pharmacological effects of SRP. Collectively, changes in *Muribaculaceae*, *Blautia*, *Colidextribacter*, and other differential taxa indicate that SRP treatment was associated with the partial shifts in the composition of the DSS-disturbed/associated microbial community. These microbial changes occurred alongside reduced inflammatory gene expression, improved histopathological injury, and alterations in fecal metabolic profiles. However, because 16S rRNA sequencing primarily provides taxonomic information rather than direct functional evidence, the present findings cannot ascertain the metabolic or immunological functions of the altered taxa [104]. Changes in microbial composition and fecal metabolic profiles were observed in parallel during SRP treatment. However, no formal cross-omics correlation analysis was conducted between differential bacterial taxa and fecal metabolites in this study. Therefore, these parallel changes should not be interpreted as direct evidence of microbiota–metabolite relationships or coordinated regulation involving inflammation, barrier-associated changes, microbial composition, and metabolism. Further research utilizing larger paired datasets and integrated microbiome–metabolome analyses, along with targeted microbial and metabolite validation, is needed to clarify these potential relationships. Additionally, metagenomic and targeted microbial-metabolite analyses will be crucial to elucidate the functional significance of these microbial changes. Equally importantly, given the limited number of biological replicates, these microbiome findings should be considered exploratory taxonomic associations and warrant validation in larger, independent cohorts.

Several limitations should be acknowledged. First, while DSS-induced colitis is commonly used to evaluate anti-inflammatory and intestinal protective activity, the present model mainly represents acute inflammatory injury and does not fully replicate the chronic and relapsing characteristics of human UC [105,106]. Furthermore, SRP administration was initiated concurrently with DSS exposure, meaning that this study assessed protective effects during disease induction rather than therapeutic efficacy once colitis had been established. Future research employing delayed-treatment protocols and chronic colitis models is necessary to further explore the therapeutic and translational potential of SRP. Second, although the extraction and ethanol-precipitation procedures were explicitly defined and the SRP yield documented, these process-level parameters do not equate to full chemical standardization. UHPLC–MS/MS provided a preliminary qualitative chemical profile of SRP, yet most constituents were tentatively annotated without confirmation using authentic standards, and the contents of representative marker compounds were not quantitatively determined. As a result, SRP cannot currently be deemed chemically standardized based on defined marker compounds, and the previously discussed compounds cannot be considered established major or active constituents. Their individual contributions to the observed protective effects also remain unknown. Future studies integrating authentic-standard-based quantification of representative marker compounds, chemical standardization, and bioactivity-guided isolation will be essential to establish a more rigorous quality-control (QC) framework for SRP and to identify the constituents that significantly contribute to its pharmacological activity. Such data would also provide a solid foundation for subsequent structure-based in silico assessments of plausible compound–target interactions [94]. Third, the evidence regarding inflammatory and inflammasome-associated transcriptional responses is limited to RT-qPCR measurements. Consequently, the present findings do not establish changes in NF-*κ*B activation status, NLRP3 inflammasome activity, protein abundance, phosphorylation, nuclear translocation, or downstream protein processing, nor do they establish a causal role for these signaling processes in the protective effects of SRP. Validation at the protein level and functional intervention studies are necessary to address these concerns. Additionally, as RT-qPCR analysis was restricted to the high-dose group, the dose–response relationship of these transcriptional changes remains undetermined. Fourth, the differential fecal metabolites were putatively annotated through untargeted metabolomics and were not confirmed using authentic standards or targeted quantitative analyses. Moreover, these metabolites may originate from host metabolism, microbial activity, dietary sources, or interactions among these factors. Therefore, interpretations regarding the biological functions of individual metabolites and enriched pathways should be considered associative and hypothesis-generating. Fifth, several downstream analyses—including histology, immunohistochemistry/immunofluorescence, RT-qPCR, metabolomics, and microbiome profiling—were conducted using a subset of three mice from each relevant group within the original cohort. Random selection was implemented to minimize investigator-driven selection bias; however, it does not overcome the limitation associated with the small number of biological replicates. This limited subset size may not adequately capture within-group biological heterogeneity, may increase sampling variability, and may reduce the precision and reproducibility of the resulting estimates. This limitation is particularly important for the metabolomic and microbiome analyses. Accordingly, these omics findings should be regarded as exploratory and require validation in larger independent cohorts. Furthermore, no functional intervention experiments were performed to ascertain whether the observed microbiota and metabolite alterations causally contributed to the protective effects of SRP. Thus, causal relationships among microbiota changes, metabolic alterations, and the observed protective effects of SRP cannot be established based on the current data. Sixth, the evaluation of epithelial barrier-related changes was restricted to the expression levels of Claudin-1, Occludin, and ZO-1, without performing functional intestinal permeability measurements or utilizing an isolated epithelial model. Consequently, the present study cannot demonstrate comprehensive or functional restoration of the intestinal barrier, nor can it determine if the observed increases in Claudin-1 and ZO-1 indicate a direct epithelial-protective effect of SRP, a secondary improvement resulting from reduced inflammatory and histopathological injury, or contributions from both processes. Additional junction-associated markers, functional permeability assays, and epithelial-cell-based studies are necessary to address these issues. Seventh, only male mice were included in the present study. Sex serves as a biological variable that may influence susceptibility to DSS-induced colitis, inflammatory and immune responses, and gut microbiota composition. Therefore, the findings of this study cannot be generalized to female mice, and potential sex-dependent differences in the protective effects of SRP were not assessed. Future studies should incorporate both male and female animals to investigate whether the efficacy and microbiota-associated responses to SRP are sex dependent. Finally, although 5-ASA was included as a positive control, the study was not designed for a direct comparative efficacy evaluation between SRP and conventional therapy. Additionally, regarding safety, while the documented food use of *R. davurica* fruits and the existence of a local food-safety standard provide contextual evidence of human exposure to this species, these data cannot be directly extrapolated to the safety of the concentrated root-derived SRP investigated here. The present study primarily aimed to evaluate efficacy rather than systemic safety. Given that all SRP-treated mice were concurrently exposed to DSS, the available body-weight and general clinical observations cannot differentiate the intrinsic tolerability of SRP from changes secondary to amelioration of colitis. Furthermore, a healthy SRP-treated control group and dedicated safety endpoints, including hematological or serum biochemical indices and histopathological evaluations of major extraintestinal organs, were not included. Thus, the systemic safety profile of SRP remains to be established in future studies.

**Figure 12:**
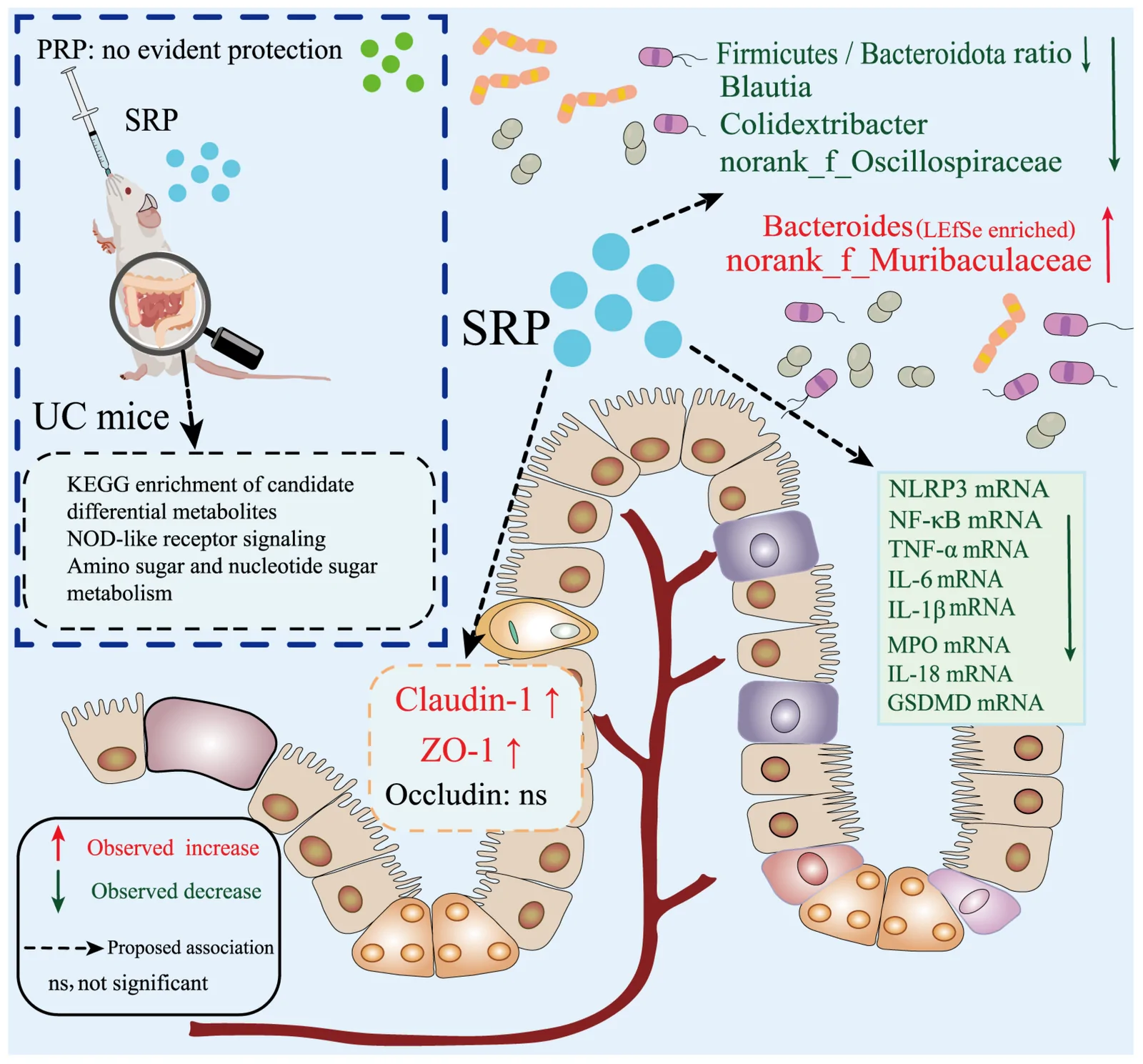
Schematic summary of SRP-associated changes in DSS-induced acute colitis mice.

In this study, SRP derived from the aqueous root extract of *R. davurica* demonstrated protective activity against the development and severity of DSS-induced acute colitis under a concurrent-intervention design. SRP treatment was associated with reduced inflammatory injury, partial improvements in barrier-associated protein expression, and alterations in gut microbiota composition and fecal metabolic profiles (Figure 12). These parallel observations provide preliminary pharmacological and associative evidence but do not confirm coordinated regulation or causal relationships among these measured processes. Further research focusing on chemical characterization, molecular mechanisms, safety evaluations, and chronic colitis models is warranted to elucidate the pharmacological and translational potential of SRP.

## 4. Materials and Methods

### 4.1. Plant Materials, Chemicals and Reagents

The roots of *R. davurica* used in this study were purchased from Jiagedaqi District, Oroqen Autonomous Banner, Hulunbuir, Inner Mongolia Autonomous Region, China, on 29 July 2024. The geographic coordinates of the source locality were approximately 50°20′14.67″ N, 124°11′56.37″ E, at an elevation of roughly 466.7 m. Botanical authentication was performed by Professor Ruifeng Fan from the School of Pharmacy, Heilongjiang University of Chinese Medicine, via examination of retained botanical materials from the same batch, including flowers, fruits, and roots. The material was confirmed as *R. davurica* (*Rosaceae*). A voucher specimen of the root material (No. RD-R-2024-0729-01) is preserved in the Laboratory of Chemistry of Chinese Materia Medica, Heilongjiang University of Chinese Medicine, Harbin, China. Retained flower and fruit reference specimens from the same batch are also housed in the laboratory, with their corresponding reference numbers provided in the Supplementary Materials.

Dextran sulfate sodium (DSS) was sourced from Dalian Meilun Biotechnology Co., Ltd. (Dalian, China), while mesalazine sustained-release granules were obtained from Shanghai Ethypharm Pharmaceutical Co., Ltd. (Shanghai, China). Sodium carboxymethyl cellulose (CMC-Na) was purchased from Shanghai Aladdin Biochemical Technology Co., Ltd. (Shanghai, China), and a 4% paraformaldehyde fixative solution was procured from Beijing Lanjiake Technology Co., Ltd. (Beijing, China). Hematoxylin and eosin (HE) staining kits, along with immunohistochemistry-related reagents, were acquired from Sigma-Aldrich, Inc. (St. Louis, MO, USA), Wuhan Servicebio Technology Co., Ltd. (Wuhan, China). Primary antibodies against tight junction proteins, including anti-Claudin-1, anti-Occludin, and anti-ZO-1, were obtained from Jiangsu Qinke Biological Research Center Co., Ltd. (Jiangsu, China), Abcam (Shanghai) Trading Co., Ltd. (Shanghai, China), and Wuhan Servicebio Technology Co., Ltd. (Wuhan, China), respectively. HRP-conjugated and FITC-conjugated secondary antibodies, along with DAPI staining reagent, were supplied by Wuhan Servicebio Technology Co., Ltd. (Wuhan, China). TRNzol Universal was purchased from Tiangen Biotech (Beijing) Co., Ltd. (Beijing, China), and the RevertAid First Strand cDNA Synthesis Kit was obtained from Thermo Fisher Scientific (Waltham, MA, USA). The 2× SYBR Green qPCR Master Mix and 96-well reaction plates were purchased from Selleck Chemicals LLC (Houston, TX, USA).

### 4.2. Preparation of WRP and the Ethanol-Precipitation Fractions SRP and PRP

Dried roots of *R. davurica* were ground into a fine powder. For the preparation of the total root aqueous extract (WRP), 100 g of the powdered material underwent reflux extraction with distilled water for 2 hours at a solid-to-liquid ratio of 1:10 (*w*/*v*). The residual plant material was extracted twice more following the same protocol. The filtrates from all extraction steps were combined, concentrated under vacuum, and freeze-dried to yield WRP, with an extraction yield of 14.24% ± 0.75% (*w*/*w*).

For the preparation of the supernatant fraction (SRP) and crude polysaccharide precipitate fraction (PRP), a separate 100 g portion of the powdered material was processed using the same aqueous extraction protocol. The resultant combined aqueous extract was evaporated to approximately 100 mL at 55 °C under vacuum, followed by the addition of four volumes of 80% ethanol. The mixture was then incubated overnight at 4 °C. After centrifugation, the liquid phase was collected, filtered, further concentrated, and freeze-dried to produce SRP, with a yield of 12.95% ± 0.66% (*w*/*w*). The precipitated fraction was redissolved in distilled water, concentrated, and subsequently freeze-dried to yield PRP, with a yield of 1.29% ± 0.16% (*w*/*w*).

### 4.3. UHPLC-Q Exactive Plus-MS/MS Conditions

The chemical constituents of SRP were analyzed using a Vanquish UHPLC system coupled with a Q Exactive Plus mass spectrometer, following a previously reported method with minor modifications [107,108]. Detailed sample-preparation procedures, chromatographic conditions, gradient-elution settings, and mass-spectrometric parameters are outlined in Table 3. Level 2 annotations were assigned when the proposed compound structure was supported by accurate mass measurements, MS/MS fragmentation patterns, chromatographic retention behavior, and comparison with spectral libraries, databases, and/or published reference data. However, these identities were not confirmed by direct comparison with an authentic reference standard analyzed under the same conditions. Proposed compound identities detected in both positive- and negative-ionization modes were cross-reviewed and included once in the final nonredundant chemical profile.

### 4.4. Animal Experimental Design

The Animal Ethics and Welfare Committee of Heilongjiang University of Chinese Medicine reviewed and approved the animal study protocol (Approval No. 2024062803). Male BALB/c mice weighing 18–22 g were sourced from Liaoning Changsheng Biotechnology Co., Ltd. (Benxi, China) under license No. SCXK (Liao) 2020-0001. All mice were housed under SPF conditions at a temperature of 22–24 °C with 40–50% relative humidity and a 12-hour light/dark cycle; standard chow and drinking water were provided ad libitum. After a 7-day acclimation period, the mice underwent the experimental procedures.

A total of 90 mice were randomly assigned to nine groups, with 10 mice in each group: normal control group (CON), model group (MOD), positive control group (PC, 400 mg/kg/day mesalazine), WRP high-dose group (WH), WRP low-dose group (WL), SRP high-dose group (SH), SRP low-dose group (SL), PRP high-dose group (PH), and PRP low-dose group (PL). The high- and low-dose levels were set at 10 and 5 g crude plant material equivalents/kg/day, respectively. Based on the corresponding preparation yields, these doses were equivalent to 1424 and 712 mg/kg/day of freeze-dried WRP, 1295 and 647.5 mg/kg/day of freeze-dried SRP, and 129 and 64.5 mg/kg/day of freeze-dried PRP for the high- and low-dose groups, respectively. The fractions were administered based on equivalent crude material rather than on equal masses of the freeze-dried preparations. Throughout the 7-day DSS administration period, mice in the CON group were given distilled water ad libitum, while all other groups received a 3% (*w*/*v*) DSS solution to establish an acute UC model. Mice in the CON and MOD groups were administered physiological saline once daily by gavage, whereas each treatment group received its corresponding drug once daily by gavage. The administration of the test fractions and 5-ASA was initiated concurrently with DSS exposure and continued throughout the 7-day induction period.

For each downstream analysis, three mice were randomly selected from each relevant experimental group from the original cohort of 10 mice, with no selection based on disease severity, colon length, or subsequent experimental outcomes. No animals were excluded from the primary in vivo efficacy analyses after group allocation.

### 4.5. DAI Evaluation

Daily monitoring throughout the experiment included assessments of body weight, stool consistency, and rectal bleeding. The DAI was calculated as the sum of three component scores, resulting in a total score between 0 and 12: (a) body weight loss, scored as 0 = < 1%, 1 = 1–5%, 2 = 5–10%, 3 = 10–15%, and 4 = ≥15%; (b) stool consistency, scored as 0 = normal, 1 = between normal and loose stool, 2 = loose stool, 3 = between loose stool and diarrhea, and 4 = diarrhea; and (c) bleeding, evaluated using occult blood test strips based on guaiac, with color intensity scored as 0 = negative (no color change), 1 = weakly positive (faint blue), 2 = positive (moderate blue), 3 = strongly positive (deep blue), and 4 = gross bleeding (visible blood without strip detection). Complete DAI scores, based on all three components, were available from days 1 to 8 and utilized for longitudinal analysis.

### 4.6. Sample Collection and Histopathology

Histopathological analysis involved three randomly selected mice per group as previously mentioned. After collection, distal colon specimens were immersed in 4% paraformaldehyde and subsequently processed for paraffin embedding. Following embedding, the samples were sectioned and subjected to HE staining. Histopathological was independently evaluated by two investigators blinded to group allocation, employing a predefined semiquantitative scoring system. The total score was computed as the sum of three parameters: (a) inflammatory severity, scored as 0 (none), 1 (mild), 2 (moderate), and 3 (severe); (b) extent of inflammatory infiltration, scored as 0 (none), 1 (mucosa), 2 (mucosa and submucosa), 3 (muscularis), and 4 (serosa); and (c) lesion area, scored as 0 (none), 1 (0–25%), 2 (>25–50%), 3 (>50–75%), and 4 (>75%). The total histological score thus ranged from 0 to 11.

### 4.7. Immunohistochemistry

For immunohistochemistry, paraffin-embedded distal colon sections were subjected to deparaffinization followed by rehydration through a graded ethanol series. Antigen retrieval was performed using a microwave in citrate buffer for 20 minutes. Following PBS washing, endogenous peroxidase activity was inhibited with 3% hydrogen peroxide in the dark for 25 minutes. The sections were then incubated with 3% bovine serum albumin for 20 minutes at room temperature to block nonspecific binding.

Primary antibodies against Claudin-1 (Affinity, AF0127, 1:400) or Occludin (Abcam, ab216327, 1:400) were applied and incubated overnight at 4 °C. After washing with PBS, HRP-conjugated goat anti-rabbit secondary antibody (Servicebio, GB23303, 1:100) was used, followed by incubation at 37 °C for 30 minutes. Immunoreactivity was visualized using a DAB substrate kit (ZSGB-BIO, ZL1-9018, 1:20), after which the sections were counterstained with hematoxylin, dehydrated, cleared with xylene, and mounted with neutral balsam.

Image acquisition was performed using a digital microscope imaging system (Motic, BA400Digital). Each section was initially surveyed at low magnification, and three non-overlapping fields were subsequently captured based on a standardized selection procedure across all sections. For blinded quantitative analysis, image files were anonymized using coded identifiers ensuring that the investigator was unaware of group allocations until quantification was complete. Claudin-1 and Occludin expression were quantified using HALO software (Indica Labs, version 101-WL-HALO-1) and expressed as the percentage of DAB-positive tissue area. Consistent threshold and analysis parameters were applied to all images, while tissue folds, tears, loss, edge artifacts, and non-tissue backgrounds were excluded from quantitative analysis.

### 4.8. Immunofluorescence Staining

For immunofluorescence staining, deparaffinization, rehydration, and antigen retrieval of paraffin-embedded distal colon sections followed the previously described protocols. After PBS washing, sections were blocked with 3% BSA for 30 minutes at room temperature and incubated overnight at 4 °C with an anti-ZO-1 antibody (Servicebio, GB115686, 1:200). A FITC-conjugated goat anti-rabbit secondary antibody (Servicebio, GB22303, 1:100) was applied at 37 °C for 30 minutes in the dark, followed by DAPI (Servicebio, G1012) counterstaining for 10 minutes and mounting with antifade medium.

Fluorescence images were captured using an Olympus VS200 slide scanner. Each section was initially surveyed at low magnification, and three non-overlapping fields were acquired at 100× and 200× magnification following the same standardized selection procedure. Quantitative analysis was conducted on the 200× images using ImageJ 1.54. For blinded quantitative analysis, image files were anonymized with coded identifiers, ensuring the investigator remained unaware of group allocations until quantification was complete. ZO-1 expression was assessed as the mean fluorescence intensity of the green channel, with identical image-analysis settings applied across all experimental groups. Tissue folds, tears, loss, edge artifacts, strong nonspecific fluorescence, and non-tissue backgrounds were excluded from quantitative analysis.

### 4.9. RT-qPCR

RT-qPCR was conducted on colonic tissues from three mice per group (*n* = 3) in the CON, MOD, and SH groups, selected according to the sampling procedure detailed in Section 4.4, following previously reported methods with minor modifications [109,110]. Total RNA was extracted from fresh colonic tissues using TRNzol Universal reagent and reverse-transcribed with the RevertAid First Strand cDNA Synthesis Kit. The resulting cDNA was amplified on an ABI StepOnePlus system using SYBR Green Master Mix and the primers listed in Table 4. Amplification consisted of an initial denaturation at 94 °C for 30 seconds, followed by 40 cycles at 94 °C for 5 seconds and 60 °C for 30 seconds. Product specificity was confirmed by melting-curve analysis, and relative mRNA expression was calculated using the 2^−ΔΔCt^ method with GAPDH as the reference gene [111].

### 4.10. Metabolomic Analysis of Fecal Samples

Fecal samples from six mice per group, randomly selected from the original experimental cohort, were analyzed using a previously reported UHPLC–HRMS/MS workflow with minor modifications [112,113]. Fecal samples stored at −80 °C were extracted with methanol/water (4:1, *v*/*v*) containing L-2-chlorophenylalanine as an internal standard. After low-temperature homogenization, sonication, precipitation, and centrifugation, the supernatants were analyzed using an ACQUITY UPLC CSH C18 column coupled to a high-resolution mass spectrometer operating in positive- and negative-ion data-dependent acquisition modes. A pooled QC sample was prepared by combining equal aliquots of the processed extracts from all experimental samples and was injected after every five experimental samples to monitor analytical stability throughout the acquisition sequence. Raw LC–MS data were processed using Progenesis QI to generate the feature-intensity matrix, which was annotated against HMDB and METLIN. Prior to multivariate analysis, feature-intensity data were mean-centered and scaled to unit variance. Principal component analysis (PCA) and partial least squares discriminant analysis (PLS-DA) were performed using the ropls package in R [114]. PLS-DA model performance was evaluated using R^2^Y and Q^2^, with model validity further verified by 200-permutation testing and cross-validated analysis of variance (CV-ANOVA). Candidate metabolites were screened using a PLS-DA-derived variable importance in projection (VIP) score > 1 in conjunction with a nominal univariate *p* value < 0.05, followed by KEGG pathway annotation and enrichment analysis [115].

### 4.11. 16S rRNA Gene Sequencing of Gut Microbiota

16S rRNA gene sequencing was carried out on colonic content samples from three randomly selected mice per group (*n* = 3/group) from the original experimental cohort, following previously reported methods with minor modifications [116,117]. Total microbial DNA extracted from frozen colonic contents was supplemented with synthetic spike-in standards at known copy numbers. The V3–V4 region was amplified using barcoded primers 338F and 806R, and the resulting libraries were sequenced on an Illumina NextSeq 2000 platform. Paired-end reads underwent processing using fastp and FLASH, followed by denoising, chimera removal, and elimination of spike-in sequences.

*α*-diversity was assessed using the Sobs, ACE, Chao1, Shannon, and Simpson indices. *β*-diversity patterns were examined via PCA and Bray–Curtis-based PCoA, with group differences evaluated by PERMANOVA utilizing 999 permutations [118]. GMHI and MDI were calculated according to established methodologies [119], and differential taxa were identified by LEfSe with LDA > 4.0 and *p* < 0.05 [120].

### 4.12. Statistical Analysis

Statistical analyses were performed using SPSS version 26.0 (IBM, USA). Longitudinal body-weight changes and DAI scores from days 1 to 8 were analyzed via linear mixed-effects models. Treatment group, time, and the group × time interaction were included as fixed effects, while mouse identity was treated as a subject-level random effect to account for repeated measurements within individual animals. Time was considered a categorical factor. Where appropriate, pairwise comparisons of estimated marginal means were adjusted for multiple comparisons using Sidak’s method. Data normality was assessed with the Shapiro–Wilk test, and homogeneity of variance was evaluated using Levene’s test. Normally distributed data with equal variances were analyzed with one-way ANOVA followed by Tukey’s post hoc test. For unequal variances, Welch’s ANOVA and the Games–Howell post hoc test were employed. Nonparametric data underwent analysis with the Kruskal–Wallis test, followed by Dunn’s test with Bonferroni correction. Tukey’s, Games–Howell, and Dunn’s tests were utilized to account for multiple pairwise comparisons among groups within each endpoint. Data are presented as mean ± SD, and statistical significance was set at a two-tailed *p* < 0.05. The statistical procedures used for different data types are detailed in this section, with sample sizes and significance notation provided in the respective figure legends.

For metabolomics, candidate metabolites were screened using VIP > 1 together with a nominal *p* < 0.05. False-discovery-rate (FDR)-adjusted *p* values from the original univariate analysis were reported alongside the nominal *p* values to assess multiple testing; however, FDR adjustment was not the primary criterion for candidate-metabolite screening. Only metabolites with an FDR-adjusted *p* value (q) < 0.05 were considered statistically significant after correction for multiple testing. KEGG pathway enrichment was assessed using the hypergeometric test with Benjamini–Hochberg correction, considering an adjusted *p* value < 0.05 as statistically significant.

In the analysis of 16S rRNA sequencing, reads were normalized to the minimum sequencing depth across samples. *α*-diversity was compared using the Kruskal–Wallis test, while *β*-diversity was evaluated through principal coordinate analysis based on Bray–Curtis distances, with group differences tested using PERMANOVA (Adonis, 999 permutations). Differential taxa were identified via linear discriminant analysis effect size (LEfSe), applying an LDA score threshold of > 4.0 and *p* < 0.05. No additional multiple-testing corrections were made for α-diversity indices or LEfSe-derived taxonomic features, with sample sizes reported in the corresponding figure legends.

## 5. Conclusions

In this study, SRP derived from the aqueous root extract of *R. davurica* demonstrated protective activity against DSS-induced acute colitis under the experimental conditions used, supporting its potential importance as a bioactive fraction of the root aqueous extract. SRP demonstrated greater efficacy in alleviating disease-related manifestations and intestinal tissue injury compared with PRP. Although the qualitative chemical profile provides preliminary insights into the multicomponent nature of SRP, the findings do not allow for attribution of its protective activity to any specific constituent. SRP treatment was associated with reduced inflammatory and inflammasome-associated gene expression and partial improvement in barrier-associated protein levels. However, the RT-qPCR data do not establish NF-*κ*B activation status, NLRP3 inflammasome activity, or a causal role for these signaling processes, while the barrier-associated data do not distinguish direct epithelial protection from improvements secondary to reduced inflammatory injury. Exploratory changes in gut microbiota composition and fecal metabolic profiles were observed during SRP treatment; these observations were obtained in parallel and do not demonstrate coordinated regulation or causal relationships among the processes involved. Therefore, these findings provide pharmacological evidence supporting SRP as a potentially important bioactive fraction of the aqueous root extract, while additional mechanistic validation is necessary to establish a defined mode of action. In summary, this study offers preliminary pharmacological and associative evidence that justifies further investigation of SRP as a candidate botanical fraction for UC, rather than confirming a specific molecular mechanism. Further research is essential to define its active constituents, underlying molecular mechanisms, systemic safety, and efficacy in chronic colitis models.

## Figures and Tables

**Table 1:** Constituents tentatively annotated in SRP under positive-ionization mode by UHPLC–Q Exactive Plus–MS/MS.

No.	Tentative Identification	Formula	Neutral Mass (Da)	Detected Mass (m/z)	*tR* (min)	Fragment	Chemical Class	Ref.
1	Choline	C_5_H_13_NO	103.09899	104.10626	0.883	104.10633; 60.0802	Alkaloids	[17]
2	L-Histidine	C_6_H_9_N_3_O_2_	155.06812	156.07539	0.923	156.1002; 156.07513; 156.04065; 110.07043	Amino Acids	[18]
3	L-Proline	C_5_H_9_NO_2_	115.06241	116.0697	0.958	116.58335; 116.06971; 70.06516; 69.49429	Amino Acids	[19,20]
4	Adenine	C_5_H_5_N_5_	135.05325	136.06053	0.969	136.06053; 119.03427	Purines	[21]
5	Adenosine	C_10_H_13_N_5_O_4_	267.09395	268.10123	1.135	268.1011; 136.06055; 119.03426	Nucleosides	[22,23]
6	Nicotinamide	C_6_H_6_N_2_O	122.04704	123.05432	1.135	123.05428; 80.04932	Alkaloids	[24]
7	L-Asparagine	C_4_H_8_N2O_3_	132.05231	133.05958	1.135	133.07475; 133.05943; 74.02364	Amino Acid Derivatives	[25]
8	L-Tyrosine	C_9_H_11_NO_3_	181.07239	182.07967	1.136	165.05296; 147.04292; 136.07454; 123.04309; 119.04823; 95.04879; 91.05392	Amino Acids	[26]
9	L-Leucine	C_6_H_13_NO_2_	131.09348	132.10075	1.17	132.11217; 132.10103; 86.09618; 69.06999	Amino Acids	[27]
10	Phloroglucinol	C_6_H_6_O_3_	126.03065	127.03792	1.342	127.03797; 109.0277; 99.0436; 81.03336; 69.03359; 53.03898	Phenolic Acids	[28]
11	L-Phenylalanine	C_9_H_11_NO_2_	165.0773	166.08458	1.389	131.04796;120.07976; 103.05359	Amino Acids	[26,29]
12	3,4-Dihydroxybenzaldehyde	C_7_H_6_O_3_	138.03041	139.03769	1.889	139.03777; 111.04327; 93.03309	Phenolic Acids	[30,31]
13	Kojic acid	C_6_H_6_O_4_	142.02539	143.03267	2.104	97.028; 69.03358	Phenolic Acids	[32,33]
14	Maltol	C_6_H_6_O_3_	126.03062	127.0379	2.108	127.03793; 99.04354; 71.04921; 55.01821; 53.03894	Phenolic Acids	[34]
15	trans-3-Indoleacrylic acid	C_11_H_9_NO_2_	187.06133	188.0686	2.135	188.0686; 170.05827; 146.05878; 144.07957; 142.06396; 118.06419; 115.05344; 91.05388	Amino Acid Derivatives	[35]
16	D-(+)-Tryptophan	C_11_H_12_N_2_O_2_	204.08785	205.09513	2.17	170.05827; 159.09013; 146.05882; 132.07968	Amino Acids	[26]
17	Catechin	C_15_H_14_O_6_	290.0753	291.08258	2.41	207.0632; 165.05298; 147.04279; 139.03769; 123.04305	Flavonoids	[26]
18	4′-Methoxyacetophenone	C_9_H_10_O_2_	150.06674	151.07402	3.316	151.07401; 135.04285; 107.04842	Phenolic Acids	[36]
19	Genistein	C_15_H_10_O_5_	270.05013	271.0574	4.697	271.0571; 243.06322	Flavonoids	[37]
20	Esculetin	C_9_H_6_O_4_	178.02498	179.03226	4.701	179.03191; 133.06377; 123.04311	Phenolic Acids	[38]
21	Ellagic acid	C_14_H_6_O_8_	302.00353	303.0108	5.127	303.00977; 284.99973; 275.01541; 247.02145; 229.01117; 201.01611; 173.02153	Tannins	[39]
22	Vanillin	C_8_H_8_O_3_	152.04596	153.05324	5.146	153.05328; 153.0173; 125.05871; 93.03313; 65.03876	Phenolic Acids	[40,41]
23	Methyl cinnamate	C_10_H_10_O_2_	162.06642	163.0737	5.188	135.0428; 107.04868; 77.0387	Phenolic Acids	[42]
24	4-Coumaric acid	C_9_H_8_O_3_	164.04593	165.05321	5.338	147.04271; 119.04817; 91.05386	Phenolic Acids	[26]
25	Daidzein	C_15_H_10_O_4_	254.0555	255.06277	5.615	255.06271; 227.06831; 199.07362	Flavonoids	[37]
26	Apocynin	C_9_H_10_O_3_	166.06163	167.06891	5.743	167.06844; 149.02214; 139.0378; 123.04311; 121.10026; 111.04332; 107.08488	Phenolic Acids	[43]
27	Naringenin	C_15_H_12_O_5_	272.06569	273.07297	5.768	153.01688; 147.04282; 119.0482	Flavonoids	[26]
28	Quercetin-3-*β*-D-glucoside	C_21_H_20_O_12_	464.09072	465.09799	5.874	303.04617; 229.04738; 97.02798; 69.03362	Flavonoids	[26]
29	Taxifolin	C_15_H_12_O_7_	304.05434	305.06161	5.885	287.05173; 259.05762; 231.06323; 195.02692; 167.03221; 153.01688; 149.02214; 123.04307	Flavonoids	[26]
30	Carvone	C_10_H_14_O	150.1032	151.11047	6.362	151.1104; 151.07463; 151.03798	Terpenoids	[44]
31	Quercetin	C_15_H_10_O_7_	302.04012	303.04739	6.391	303.04593; 229.04752; 201.05254; 153.01682; 137.02216; 121.0274; 68.99715	Flavonoids	[45,46]
32	Cynaroside	C_21_H_20_O_11_	448.09627	449.10355	6.588	449.10468; 287.0513	Flavonoids	[47]
33	Phloretin	C_15_H_14_O_5_	274.08132	275.08859	7.042	169.04774; 149.05843; 107.04845	Flavonoids	[26]
34	Naringenin Chalcone	C_15_H_12_O_5_	272.06575	273.07303	7.274	153.01685; 147.04283	Flavonoids	[48]
35	Isoliquiritigenin	C_15_H_12_O_4_	256.07106	257.07834	7.84	239.06825; 147.04286; 137.02209; 119.04824; 91.05381	Flavonoids	[49]
36	4-Hydroxycoumarin	C_9_H_6_O_3_	162.03009	163.03737	8.831	163.03726; 135.04294	Phenolic Acids	[50]
37	Methyl caffeate	C_10_H_10_O_4_	194.05625	195.06352	8.838	195.02577; 163.03738	Phenolic Acids	[51]
38	Kaempferol	C_15_H_10_O_6_	286.04518	287.05246	9.021	287.05154; 153.01688	Flavonoids	[52,53]
39	Sedanolide	C_12_H_18_O_2_	176.11855	177.12582	9.61	177.12552; 107.08486; 93.06953; 79.0541	Terpenoids	[26]
40	Chrysin	C_15_H_10_O_4_	254.05542	255.0627	10.972	255.06271; 153.01686; 147.04291; 129.0323; 68.99717	Flavonoids	[54]
41	Ursolic acid	C_30_H_48_O_3_	456.35686	457.36414	12.069	457.33112; 439.31628	Terpenoids	[55]
42	9-Oxo-10(E),12(E)- octadecadienoic acid	C_18_H_30_O_3_	294.21592	295.22317	12.28	277.21307; 151.11044; 95.04875; 81.03333; 69.06995; 67.05437	Fatty Acids	[56]
43	2-Linoleoyl glycerol	C_21_H_38_O_4_	336.26335	337.27063	12.72	337.26965; 93.06944; 83.0853; 81.06968	Fatty Acids	[57]
44	Linoleoyl ethanolamide	C_20_H_37_NO_2_	323.27953	324.2868	12.929	324.2861; 109.10052; 62.06028	Fatty Acids	[58,59]
45	Oleoyl ethanolamide	C_20_H_39_NO_2_	325.29503	326.30231	13.813	326.30148; 309.27484; 83.08537	Fatty Acids	[60]
46	Coniferyl aldehyde	C_10_H_10_O_3_	178.06142	179.0687	13.816	179.06793; 161.05812; 147.0426; 133.06366; 79.05409	Phenolic Acids	[61]
47	Oleamide	C_18_H_35_NO	281.26921	282.27649	15.415	282.27591; 265.24985; 247.23993	Fatty Acids	[62]

**Table 2:** Constituents tentatively annotated in SRP under negative-ionization mode by UHPLC–Q Exactive Plus–MS/MS.

No.	Tentative Identification	Formula	Neutral Mass (Da)	Detected Mass (m/z)	*tR* (min)	Fragment	Chemical Class	Ref.
1	D-(-)-Quinic acid	C_7_H_12_O_6_	192.06091	191.05363	0.455	93.03221; 85.02718	Organic Acids	[63]
2	Arginine	C_6_H_14_N_4_O_2_	174.10919	173.10191	0.791	173.10199; 156.07529; 131.08011	Amino Acids	[64]
3	Gallic acid	C_7_H_6_O_5_	170.01893	169.01166	1.221	125.02198; 97.02709; 79.01662; 69.03241	Phenolic Acids	[65]
4	4-Anisic acid	C_8_H_8_O_3_	152.04492	151.03764	2.514	151.03769; 107.04786; 59.0118	Phenolic Acids	[49]
5	Salicylic acid	C_7_H_6_O_3_	138.02922	137.02194	2.671	137.02196; 93.03225	Phenolic Acids	[66]
6	Caffeic acid	C_9_H_8_O_4_	180.03979	179.03252	3.629	135.04269; 134.03481; 89.02208	Phenolic Acids	[67]
7	(-)-Epicatechin	C_15_H_14_O_6_	290.07713	289.06985	3.999	289.06958; 245.07997; 203.06883; 151.0377; 137.02202; 125.02198; 109.0271	Flavonoids	[68]
8	Syringic acid	C_9_H_10_O_5_	198.05034	197.04306	5.374	197.04308; 182.01956; 153.05325; 123.00642	Phenolic Acids	[69]
9	Miquelianin	C_21_H_18_O_13_	478.07088	477.0636	5.772	477.05676; 301.03217	Flavonoids	[70]
10	trans-Ferulic acid	C_10_H_10_O_4_	194.05536	193.04808	5.829	149.05843; 134.03494	Phenolic Acids	[71]
11	Luteolin	C_15_H_10_O_6_	286.04558	285.0383	5.912	285.03836; 241.04854; 175.03754	Flavonoids	[72]
12	Azelaic acid	C_9_H_16_O_4_	188.10234	187.09506	6.92	187.09508; 169.08452; 125.09472; 97.06347; 57.03252	Fatty Acids	[73]
13	Eriodictyol	C_15_H_12_O_6_	288.06151	287.05423	7.888	287.05353; 151.00117; 125.02197; 107.01143; 83.01155; 65.00116	Flavonoids	[74]
14	Abscisic acid	C_15_H_20_O_4_	264.13457	263.12729	7.897	219.13672; 204.11316; 201.12613; 151.07401; 136.05055	Terpenoids	[75,76]
15	(±)12,13-DiHOME	C_18_H_34_O_4_	314.24376	313.23648	11.603	313.23492; 129.08961	Fatty Acids	[77]
16	(±)9,10-DiHOME	C_18_H_34_O_4_	314.24354	313.23627	11.652	313.23517; 201.11066	Fatty Acids	[77]
17	12-oxo Phytodienoic Acid	C_18_H_28_O_3_	292.20189	291.19461	11.955	291.19418; 247.02271	Fatty Acids	[76]
18	(±)13-HODE	C_18_H_32_O_3_	296.23277	295.22549	12.046	295.22519; 277.21573; 195.13663	Fatty Acids	[78]
19	13(S)-HOTrE	C_18_H_30_O_3_	294.21751	293.21024	12.168	293.20975; 195.1367	Fatty Acids	[79]
20	(±)9-HpODE	C_18_H_32_O_4_	294.2177	293.21042	12.257	293.20984; 275.19995; 197.11534	Fatty Acids	[80]
21	Arachidic Acid	C_20_H_40_O_2_	312.30095	311.29367	15.34	311.29251; 311.21857; 311.16629; 183.00957	Fatty Acids	[81]

**Table 3:** UHPLC–Q Exactive Plus–MS/MS conditions for the chemical profiling of SRP.

Category	Parameter	Condition
Sample preparation	Sample form	Lyophilized SRP powder
	Reconstitution solvent	LC–MS-grade methanol
	Sample concentration	1 mg/mL
	Vortex mixing	1 min
	Centrifugation	14,000 rpm for 10 min
	Filtration	0.22 μm membrane filter
UHPLC conditions	UHPLC system	Thermo Scientific Vanquish UHPLC system
	Analytical column	Hypersil Gold Vanquish C18 column
	Column dimensions	2.1 × 100 mm, 1.9 μm
	Column temperature	30 °C
	Mobile phase A	Water containing 0.1% formic acid
	Mobile phase B	Acetonitrile
	Flow rate	0.3 mL/min
	Injection volume	5 μL
	Total run time	18 min
Gradient program	0–2 min	10% B
	2–10 min	10–50% B
	10–10.1 min	50–80% B
	10.1–13 min	80% B
	13–14 min	80–95% B
	14–14.1 min	95–10% B
	14.1–18 min	10% B
Mass spectrometry conditions	Mass spectrometer	Thermo Scientific Q Exactive Plus
	Ionization source	Electrospray ionization
	Acquisition mode	Full MS/data-dependent MS/MS
	Data-dependent mode	TopN
	Full-scan resolution	70,000
	Full-scan AGC target	1 × 10^6^
	Maximum injection time	100 ms

**Table 4:** Primer Sequences.

Name		Sequences (5′~3′)	Size (bp)
**TNF-*α***	F	CTTCCAGAACTCCAGGCGGTGC	184
	R	TTGGTGGTTTGTGAGTGTGAGGGTC	
**IL-6**	F	GAAATGATGGATGCTACCAAACTG	139
	R	ACTCTGGCTTTGTCTTTCTTGTTATC	
**IL-1*β***	F	TGAACAACAAAAAAGCCTCGTGC	201
	R	GTGGGTGTGCCGTCTTTCATTAC	
**MPO**	F	ATGCCCACCGAATGACAAGTATC	167
	R	AGCCATTGCGATTGACTCCAG	
**NF-*κ*B**	F	GAACTACGGATTTCCTCCCTACG	131
	R	CATCACTCTTGGCACAATCTTTAGG	
**NLRP3**	F	GCTAAGAAGGACCAGCCAGAGT	127
	R	TACAAATGGAGATGCGGGAGA	
**GSDMD**	F	CAGTTCCAGTGCCTCCAT	143
	R	CCACAAACAGGTCATCCC	
**IL-18**	F	AAAATGGAGACCTGGAATCAGACAA	93
	R	TGTCAACGAAGAGAACTTGGTCATT	
**GAPDH**	F	CGGTGCTGAGTATGTCGTGGAGTC	100
	R	GGCGGAGATGATGACCCTTTTG	

F: Forward sequence; R: Reverse sequence.

## Data Availability

The original contributions presented in this study are included in the article. Further inquiries can be directed to the corresponding author.

## Supplementary Materials

The following supporting information can be downloaded at: https://www.mdpi.com/article/10.3390/ph19091372/s1, Supplementary Material S1: Detailed experimental methods for the comparative pharmacological screening of flowers, fruits, and roots of *R. davurica* Pall.; Supplementary Material S2: Supplementary results and discussion on the comparative efficacy and the rationale for selecting the root aqueous extract; Figure S1: Body weight changes and DAI scores in DSS-induced colitis mice; Figure S2: Representative colon morphology and *quantitative* length analysis; Figure S3: Histopathological evaluation of distal colon tissues with HE staining and semiquantitative scores; Figure S4: Colonic inflammatory cytokine levels (TNF-*α*, IL-1*β*, and IL-6) and corresponding quantitative data. Figure S5. Longitudinal DAI scores were analyzed using a linear mixed-effects model with treatment group, time, and the group × time interaction as fixed effects and mouse identity as a subject-level random effect. Significant group, time, and group × time effects were observed (all *p* < 0.001). Table S1: Levels of inflammatory cytokines in colonic tissues of DSS-induced colitis mice (mean ± SD, pg/mg protein, n = 6). Table S2: Representative candidate differential fecal metabolites associated with DSS induction and SRP treatment.

## Abbreviations

The following abbreviations are used in this manuscript:

5-ASA	5-aminosalicylic acid (5-ASA; mesalazine)
16S rRNA	16S ribosomal RNA
ANOVA	analysis of variance
ASV	amplicon sequence variant
BSA	bovine serum albumin
CMC-Na	carboxymethyl cellulose sodium
DAB	3,3′-diaminobenzidine
DAI	disease activity index
DAPI	4′,6-diamidino-2-phenylindole
DSS	dextran sulfate sodium
ESI	electrospray ionization
FDR	false discovery rate
FITC	fluorescein isothiocyanate
GAPDH	glyceraldehyde-3-phosphate dehydrogenase
GMHI	gut microbiome health index
GSDMD	gasdermin D
HMDB	Human Metabolome Database
HRP	horseradish peroxidase
IBD	inflammatory bowel disease
IL-1*β*	interleukin-1 beta
IL-6	interleukin-6
IL-18	interleukin-18
KEGG	Kyoto Encyclopedia of Genes and Genomes
LDA	linear discriminant analysis
LEfSe	linear discriminant analysis effect size
MDI	microbial dysbiosis index
NEG	negative ionization mode
NF-*κ*B	nuclear factor kappa B
NIH	National Institutes of Health
NLRP3	NOD-like receptor family pyrin domain containing 3
PCA	principal component analysis
PCoA	principal coordinate analysis
PERMANOVA	permutational multivariate analysis of variance
PLS-DA	partial least squares discriminant analysis
POS	positive ionization mode
QC	quality control
RT-qPCR	reverse transcription quantitative polymerase chain reaction
SD	standard deviation
TCM	Traditional Chinese Medicine
TNF-*α*	tumor necrosis factor alpha
UC	ulcerative colitis
UHPLC-MS/MS	ultra-high-performance liquid chromatography–tandem mass spectrometry
VIP	variable importance in projection
UHPLC	ultra-high-performance liquid chromatography
