# Supplementary material for: Supernatants of Water Extraction–Ethanol Precipitation from Roots of Rosa davurica Pall. Ameliorate Ulcerative Colitis in Mice: From Gut Microbes to Metabolites
Source: Pharmaceuticals (Basel). 2026 Aug 30;19(9):1372. doi: 10.3390/ph19091372 (PMC13610652; doi:10.3390/ph19091372)
Supplement: Supplementary file 1 [file pharmaceuticals-19-01372-s001.zip › pharmaceuticals-4480124-supplementary.pdf]

Article

Supernatants of water extraction–ethanol precipitation from roots of *Rosa davurica* Pall. ameliorate ulcerative colitis in mice: From gut microbes to metabolites

Lihao Wang [1], Wuyou Gao [1], Yuesong Xiao [1], Ting Yang [1], Jingwei Wang [1]and, Yanping Sun [1,*]

Screening of Anti-Ulcerative Colitis Effects of Different Medicinal Parts of *Rosa davurica* Pall.

S1. Supplementary Methods

S1.1 Plant Material and Preparation of Extracts

The roots of *R. davurica* used in this study were purchased from Jiagedaqi District, Oroqen Autonomous Banner, Hulunbuir, Inner Mongolia Autonomous Region, China, on July 29, 2024. The geographic coordinates of the source locality were approximately 50°20′14.67″ N, 124°11′56.37″ E, at an elevation of roughly 466.7 m. Botanical authentication was performed by Professor Ruifeng Fan from the School of Pharmacy, Heilongjiang University of Chinese Medicine, via examination of retained botanical materials from the same batch, including flowers, fruits, and roots. The material was confirmed as *R. davurica* (*Rosaceae*). A voucher specimen of the root material (No. RD-R-2024-0729-01) is preserved in the Laboratory of Chemistry of Chinese Materia Medica, Heilongjiang University of Chinese Medicine, Harbin, China. Retained flower and fruit reference specimens from the same batch are also housed in the laboratory, with their corresponding reference numbers provided in the Supplementary Materials.

For aqueous extraction, 100 g of powdered plant material was refluxed with distilled water (1:10, w/v) at 100 °C for 2 h. The residue was re-extracted twice under identical conditions. The combined filtrates were concentrated under reduced pressure to approximately 50 mL. For ethanol extraction, 100 g of powdered material was refluxed with 75% ethanol (1:10, w/v) for 2 h, repeated three times, and concentrated to 50 mL. All extracts were stored at 4 °C until administration.

S1.2 Animals and Experimental Design

All animal procedures in this preliminary screening were approved by the Animal Ethics and Welfare Committee of Heilongjiang University of Chinese Medicine (Approval No. 2024062803). In a separate preliminary screening experiment, seventy-two male BALB/c mice (18–22 g) were purchased from Liaoning Changsheng Biotechnology Co., Ltd. (license No. SCXK (Liao) 2020-0001) and housed under SPF conditions at 22–24 °C and 40–50% relative humidity, with a 12 h light/dark cycle and free access to standard

feed and drinking water. After 7 days of acclimatization, mice were randomly divided into nine groups (n = 8 per group): normal control (CON), model (MOD), positive control (MES, 400 mg/kg/day mesalazine), flower aqueous extract (FlA, 10 g crude drug/kg), flower ethanol extract (FlE, 10 g crude drug/kg), fruit aqueous extract (FrA, 10 g crude drug/kg), fruit ethanol extract (FrE, 10 g crude drug/kg), root aqueous extract (RA, 10 g crude drug/kg), and root ethanol extract (RE, 10 g crude drug/kg).

During the 7-day experimental period, the CON group received distilled water ad libitum, whereas all other groups were given 3% (w/v) DSS solution (molecular weight 36–50 kDa; Dalian Meilun Biotechnology Co., Ltd., China) ad libitum to induce acute ulcerative colitis. The CON and MOD groups were administered physiological saline by gavage once daily, and each treatment group received the corresponding extract by gavage once daily. On day 8, mice were sacrificed by cervical dislocation after fasting for 12 h. The entire colon from the cecum to the anus was excised, photographed, and measured for length. Distal colon segments were fixed in 4% paraformaldehyde for histopathology, and the remaining colonic tissue was snap-frozen in liquid nitrogen and stored at −80 °C for cytokine analysis.

S1.3 DAI Evaluation

The DAI was calculated as the sum of three component scores, yielding a total score ranging from 0 to 12: (i) body weight loss: 0 = <1%, 1 = 1–5%, 2 = 5–10%, 3 = 10–15%, 4 = ≥15%; (ii) stool consistency: 0 = normal, 1 = between normal and loose stool, 2 = loose stool, 3 = between loose stool and diarrhea, 4 = diarrhea; and (iii) bleeding: 0 = negative, 1 = weakly positive, 2 = positive, 3 = strongly positive, and 4 = gross bleeding. Higher total scores indicated greater disease activity.

S1.4 Histopathological Analysis

Paraffin-embedded distal colon sections (4 μm) were stained with HE. Histological damage was evaluated by two independent observers blinded to group assignment using a validated semiquantitative scoring system (0–4) as follows: 0 = normal morphology with no inflammation; 1 = partial goblet cell loss and mild inflammatory infiltration; 2 = extensive goblet cell loss and moderate mucosal inflammation; 3 = partial crypt loss, extensive mucosal and submucosal inflammatory infiltration with mucosal edema; 4 = extensive crypt destruction and widespread transmural inflammation.

Note: The semiquantitative scoring system used in this preliminary screening (range 0--4) was a simplified version adapted for rapid pharmacological evaluation. The formal experiment reported in the main text employed a more granular 0--11 scoring system to capture detailed histopathological changes.

S1.5 Cytokine Measurement by ELISA

Colonic tissue homogenates were prepared in cold PBS. The protein concentration was determined by the BCA method. Levels of tumor necrosis TNF-α, IL-1β, and IL-6 were

measured using commercial ELISA kits (catalog Nos. JL10484, JL18442, and JL20268, respectively; Shanghai Jianglai Biological Technology Co., Ltd., China) according to the manufacturer's instructions. All samples were assayed in duplicate.

S1.6 Statistical Analysis

Data are presented as mean ± SD. Normality and homogeneity of variance were assessed by the Shapiro–Wilk and Levene tests, respectively. For parametric data, ANOVA followed by Tukey's multiple comparisons test was used. For non-normally distributed data or ordinal scores, the Kruskal–Wallis H test was applied, followed by Dunn's test with Bonferroni correction. A two-tailed $p < 0.05$ was considered statistically significant, and $p < 0.01$ was considered highly significant. All analyses were performed using SPSS 26.0 (IBM, USA).

S2 Supplementary Results

S2.1 Effects on Body Weight and Disease Activity Index

During the 7-day experimental period, mice in the CON group exhibited normal mental status, bright and smooth fur, and a gradual increase in body weight. In contrast, mice in the MOD group displayed obvious abnormalities from day 3 onward, including lethargy, hunched posture, rough and dull fur, reduced food and water intake, and significant body weight loss. Compared with the MOD group, mice treated with mesalazine (MES), RA, RE, FrA, and FrE showed varying degrees of body weight recovery, whereas FlA and FlE did not produce obvious improvement (Fig S1A).

The DAI score in the MOD group was significantly elevated compared with the CON group ($p < 0.01$). Treatment with MES, RA, and RE significantly reduced DAI scores relative to the MOD group (all $p < 0.05$). No significant changes in DAI were observed in the FlA, FlE, FrA, or FrE groups compared with the MOD group (Fig S1B).

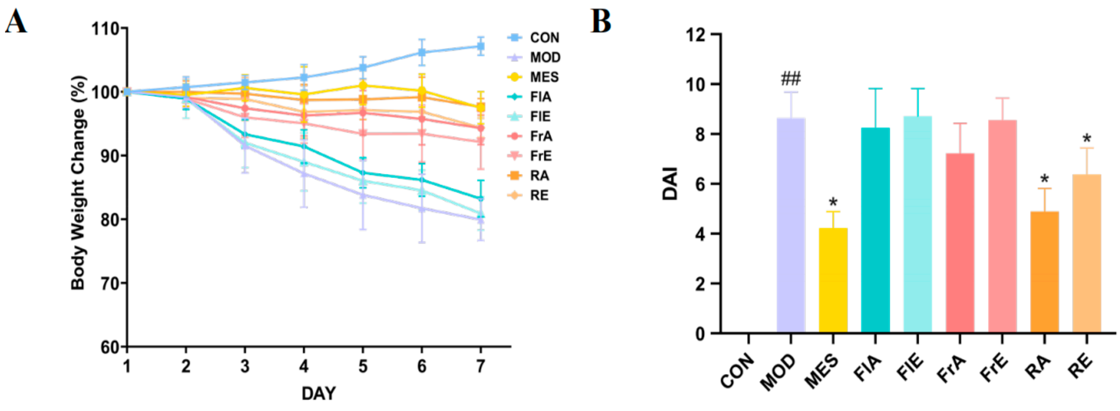

Figure S1. Effects of *R. davurica* extracts on body weight and disease activity in DSS-induced acute colitis mice. (A) Body weight change (%) over the 7-day experimental period (n = 8). (B) DAI scores at day 7 (n = 8). Data are presented as mean ± SD. $^{\#}p < 0.05$, $^{\#\#}p < 0.01$ compared with the CON group; $^{*}p < 0.05$, $^{**}p < 0.01$ compared with the MOD group.

S2.2 Effects on Colon Length

The mean colon length in the CON group was 6.99 ± 0.36 cm. DSS induction significantly shortened the colon to 4.64 ± 0.90 cm in the MOD group ($p < 0.01$ vs. CON). Administration of MES (6.06 ± 0.34 cm) and RA (6.17 ± 0.83 cm) significantly ameliorated colon shortening (both $p < 0.01$ vs. MOD). FrA and RE also exhibited a modest but significant increase in colon length compared with the MOD group ($p < 0.05$). FlA and FlE did not significantly affect colon length (Fig S2).

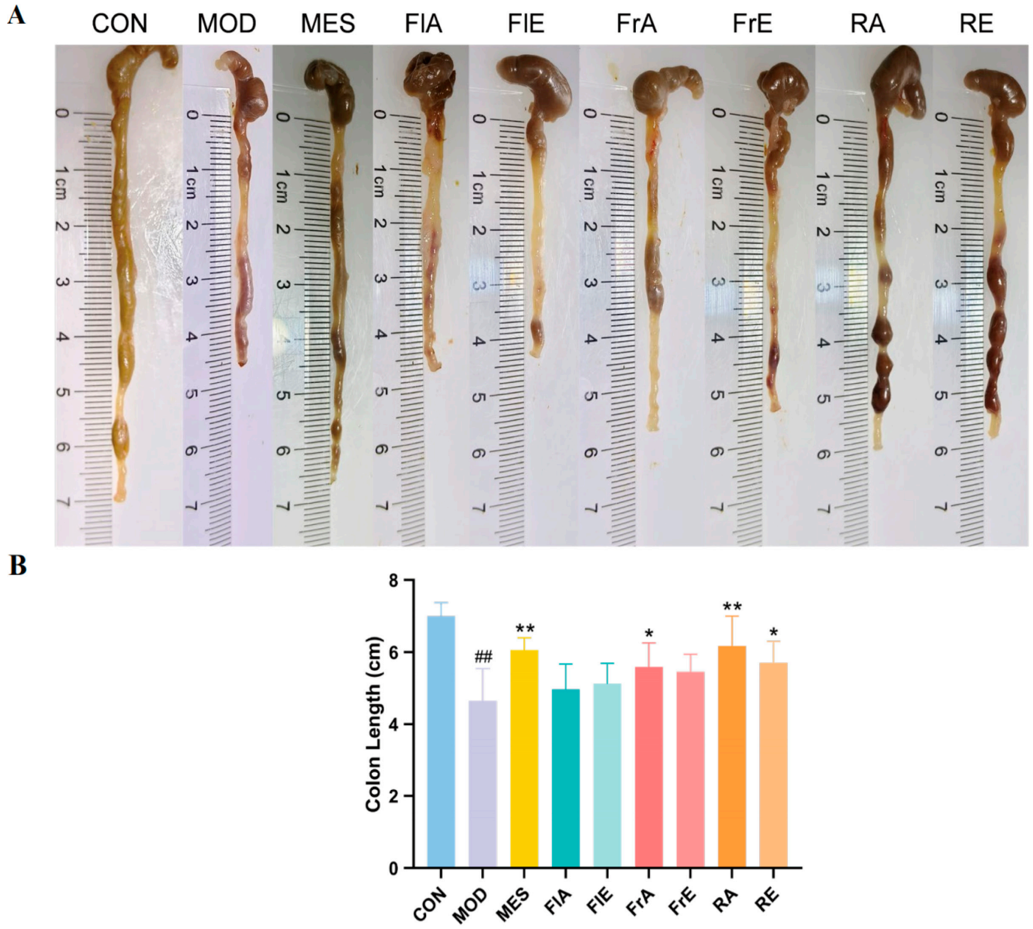

Figure S2. Representative colon morphology and quantitative length analysis. (A) Representative photographs of colon segments from each group. (B) Colon lengths at necropsy. Data are presented as mean ± SD (n = 8). $^{\#}p < 0.05$, $^{\#\#}p < 0.01$ compared with the CON group; $^{*}p < 0.05$, $^{**}p < 0.01$ compared with the MOD group.

S2.3 Histopathological Evaluation

HE staining revealed intact colonic architecture in the CON group, with closely arranged epithelial cells, regular crypt architecture, orderly intestinal glands, and abundant goblet cells. The MOD group showed severe disruption of tissue structure, including extensive mucosal damage, ulceration, massive inflammatory cell infiltration, and widespread crypt destruction, resulting in a significantly elevated histopathological score ($p < 0.01$ vs. CON). Among all extracts tested, only RA significantly reduced the histopathological score compared with the MOD group ($p < 0.05$). MES also significantly improved histological damage ($p < 0.05$ vs. MOD). The remaining treatment groups (FlA,

FlE, FrA, FrE, RE) showed some morphological improvement but did not reach statistical significance versus the MOD group (Fig S3).

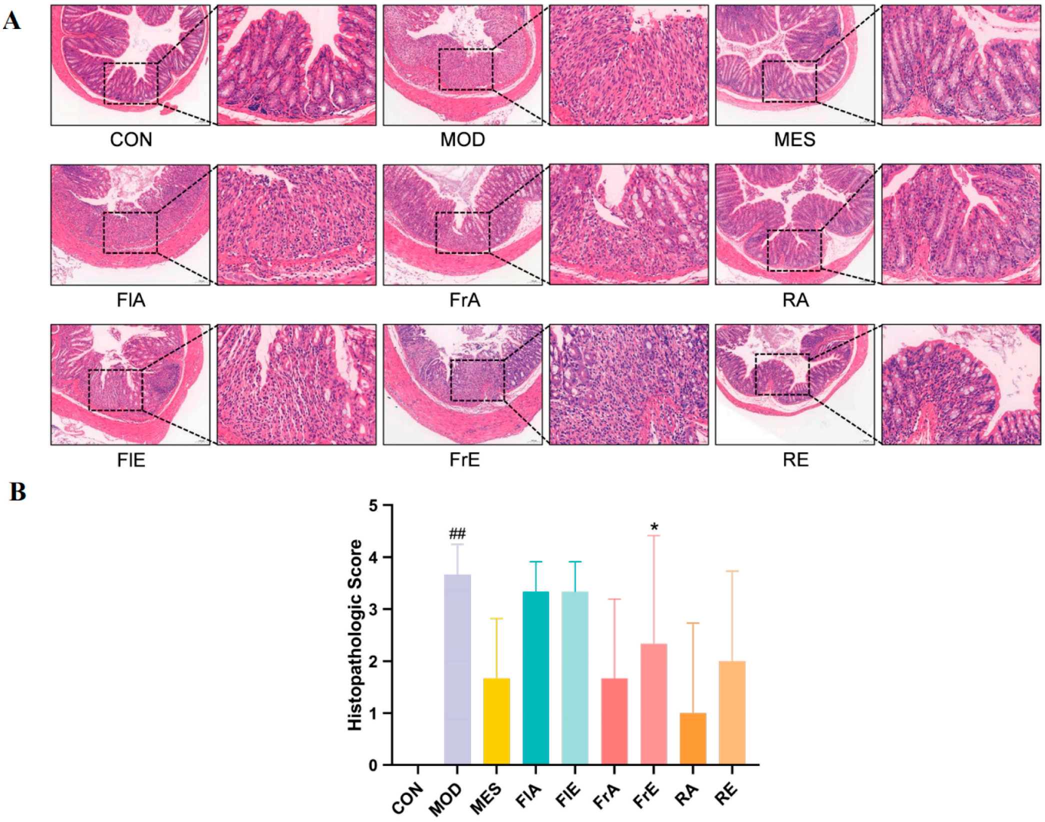

Figure S3. Histopathological evaluation of distal colon tissues. (A) Representative HE-stained sections (100×, with 400× magnified insets). (B) Semiquantitative histopathological scores (range 0 – 4). Data are shown as mean ± SD (n = 3). $^{\#}p < 0.05$, $^{\#\#}p < 0.01$ compared with the CON group; $^{*}p < 0.05$, $^{**}p < 0.01$ compared with the MOD group.

S2.4 Effects on Colonic Inflammatory Cytokines

Compared with the CON group, colonic levels of TNF-α, IL-1β, and IL-6 were markedly elevated in the MOD group (all $p < 0.01$), confirming successful establishment of the inflammatory model. MES significantly suppressed all three cytokines relative to the MOD group (all $p < 0.01$). RA significantly reduced TNF-α ($p < 0.01$), IL-1β ($p < 0.05$), and IL-6 ($p < 0.01$) compared with the MOD group. RE significantly decreased TNF-α and IL-1β (both $p < 0.05$) but did not significantly reduce IL-6. FrA significantly lowered TNF-α and IL-1β (both $p < 0.05$) without significantly affecting IL-6. FlA, FlE, and FrE did not produce significant reductions in any of the three cytokines compared with the MOD group (Table S1, Fig S4).

Table S1. Levels of inflammatory cytokines in colonic tissues of DSS-induced colitis mice (mean ± SD, pg/mg protein, n = 6).

Group	TNF-α	IL-1β	IL-6

CON	27.57 ± 3.39	72.79 ± 9.41	38.96 ± 20.40
MOD	80.78 ± 7.05$^{\#\#}$	300.36 ± 16.44$^{\#\#}$	198.08 ± 41.67$^{\#\#}$
MES	50.77 ± 2.86**	110.30 ± 9.19**	114.74 ± 21.31**
FlA	78.74 ± 9.51	291.18 ± 24.13	194.62 ± 17.37
FlE	79.73 ± 6.38	303.27 ± 20.49	177.51 ± 37.05
FrA	71.52 ± 6.70*	266.55 ± 25.25*	179.01 ± 26.77
FrE	74.70 ± 7.92	281.96 ± 32.28	193.59 ± 30.26
RA	55.14 ± 6.70**	159.79 ± 36.53*	132.38 ± 11.60**
RE	66.04 ± 6.87*	197.30 ± 12.85*	159.14 ± 11.96

$^{\#}p < 0.05$, $^{\#\#}p < 0.01$ compared with the CON group;
$^{*}p < 0.05$, $^{**}p < 0.01$ compared with the MOD group.

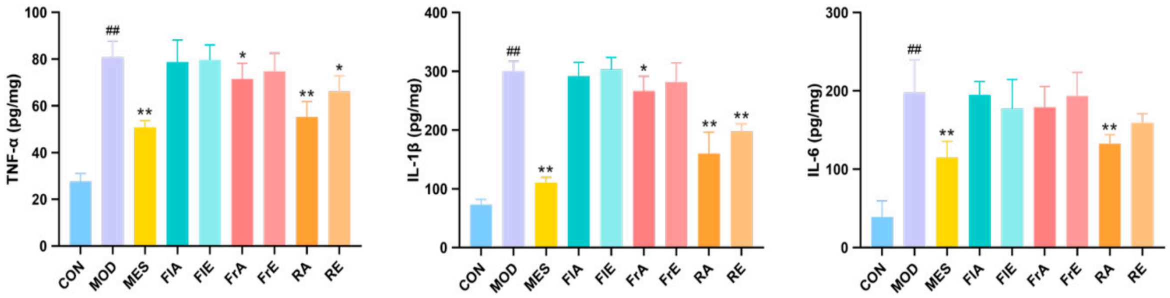

Figure S4. Colonic inflammatory cytokine levels measured by ELISA. (A) TNF-α. (B) IL-1β. (C) IL-6. Data are shown as mean ± SD (n = 6). $^{\#}p < 0.05$, $^{\#\#}p < 0.01$ compared with the CON group; $^{*}p < 0.05$, $^{**}p < 0.01$ compared with the MOD group.

Supplementary Discussion

A preliminary comparative screening was performed to evaluate the anti-UC potential of the aqueous and ethanol extracts from three medicinal parts of *R. davurica* Pall. As detailed in the Supplementary Results, neither the FlA nor the FlE significantly ameliorated DSS-induced body weight loss, DAI scores, colon shortening, histopathological damage, or colonic pro-inflammatory cytokine levels under the present experimental conditions. The fruit extracts showed limited efficacy: the FrA significantly restored colon length and reduced TNF-α and IL-1β levels, whereas the FrE only modestly increased colon length without significantly improving DAI, histopathological injury, or cytokine profiles. By contrast, the root aqueous extract (RA) demonstrated the most comprehensive and robust therapeutic profile among all tested groups, significantly reducing DAI scores, attenuating colon shortening, ameliorating mucosal histopathological damage, and suppressing all three measured pro-inflammatory cytokines. Although the root RE also significantly improved certain endpoints (DAI, colon length, TNF-α, and IL-1β), it failed to significantly attenuate histopathological damage or IL-6 levels. Collectively, these comparative, data-driven results objectively identify the root aqueous extract as the most potent anti-UC starting material among the evaluated parts and solvent systems,

thereby providing the rationale for its selection in subsequent bioassay-guided fractionation into the PRP and SRP fractions.

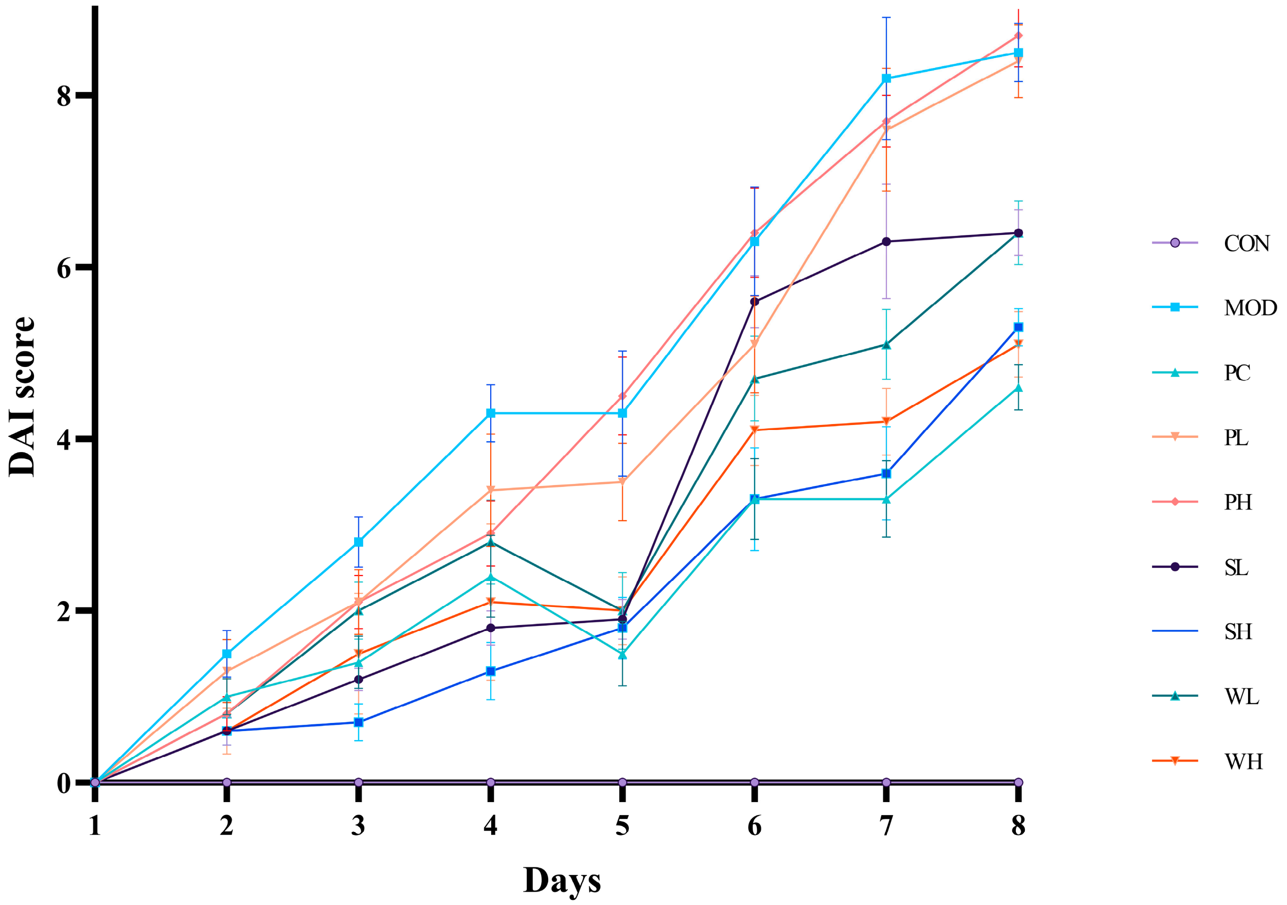

Figure S5. Longitudinal changes in disease activity index (DAI) scores from days 1 to 8 in DSS-induced acute colitis mice. Data are presented as mean ± SD (n = 10 mice/group). Longitudinal DAI scores were analyzed using a linear mixed-effects model with treatment group, time, and the group × time interaction as fixed effects and mouse identity as a subject-level random effect. Significant effects of treatment group, time, and the group × time interaction were observed (all $p < 0.001$).

Table S2. Representative candidate differential fecal metabolites associated with DSS induction and SRP treatment.

No.	Putatively annotated metabolite	Ion mode	m/z	RT (min)	Formula	MOD vs. CON	SRP vs. MOD	FC (MOD/SRP)	PLS-DA VIP	Nominal p	FDR-adjusted p (q)	Representative KEGG pathway(s)
1	10,11-Dihydro-12R-hydroxy-leukotriene E4	Positive	499.286854	3.351	$C_{23}H_{39}NO_6S$	↑***	↓#	1.2018	1.4221	0.04361	0.1612	—
2	D-Glucosaminide	Positive	506.191786	3.505	$C_{18}H_{35}N_3O_{13}$	↑***	↓###	1.3007	2.3497	0.0007619	0.01755	Amino sugar and nucleotide sugar metabolism
3	Homocysteine	Negative	180.032957	1.241	$C_4H_9NO_2S$	↓***	↑##	0.9257	1.2546	0.004077	0.04250	Sulfur metabolism; cysteine and methionine metabolism; NOD-like receptor signaling pathway
4	5-Phosphoribosylamine	Negative	228.027449	4.163	$C_5H_{12}NO_7P$	↑***	↓##	1.0869	1.1289	0.004955	0.04741	Alanine, aspartate and glutamate metabolism; purine metabolism
5	γ-Glu-Leu	Positive	261.144119	3.141	$C_{11}H_{20}N_2O_5$	↓***	↑##	0.9410	1.0642	0.004228	0.04345	—
6	γ-Glutamylvaline	Positive	247.128581	2.268	$C_{10}H_{18}N_2O_5$	↓***	↑##	0.9430	1.0966	0.001270	0.02355	—
7	Histidylvaline	Positive	255.144846	2.609	$C_{11}H_{18}N_4O_3$	↑***	↓#	1.3586	2.3225	0.01090	0.07219	—
8	Pro-Pro	Positive	254.149637	2.780	$C_{10}H_{16}N_2O_3$	↑***	↓##	1.2293	2.0692	0.003408	0.03931	—
9	N-Lactoylvaline	Negative	188.092238	1.857	$C_8H_{15}NO_4$	↑***	↓##	1.1311	1.4003	0.002677	0.03453	—
10	Indole-3-carboxylic acid O-sulfate	Negative	286.002656	2.956	$C_9H_7NO_5S$	↑***	↓###	1.0865	1.3962	8.381×10^{-5}	0.004111	—
11	Behenic acid	Positive	379.294848	7.944	$C_{22}H_{44}O_2$	↑**	↓##	1.1828	1.4137	0.003503	0.03972	Biosynthesis of unsaturated fatty acids
12	L-Argininium(1+)	Positive	176.128027	0.960	$C_6H_{15}N_4O_2^+$	↓***	↑#	0.8239	1.8296	0.02027	0.1049	—

Notes: Metabolites were putatively annotated by untargeted LC–MS/MS without authentic-standard confirmation. CON corresponds to NC, and SRP to RDRH/SH in the original metabolomics output. FC was calculated as MOD/SRP. Candidate metabolites were screened using VIP > 1 and nominal $p < 0.05$; FDR-adjusted q values for the MOD-versus-SRP comparison are reported separately, with q < 0.05 considered statistically significant after multiple-testing correction. For MOD vs. CON: $^{*}p < 0.05$, $^{**}p < 0.01$, $^{***}p < 0.001$; for SRP vs. MOD: $^{\#}p < 0.05$, $^{\#\#}p < 0.01$, $^{\#\#\#}p < 0.001$. KEGG enrichment was assessed using the hypergeometric test with Benjamini–Hochberg correction. n = 3 biological samples/group.
